# Thromboembolic Disease and Cardiac Thrombotic Complication in COVID-19: A Systematic Review

**DOI:** 10.3390/metabo12100889

**Published:** 2022-09-22

**Authors:** Francesco Nappi, Pierluigi Nappi, Ivancarmine Gambardella, Sanjeet Singh Avtaar Singh

**Affiliations:** 1 Department of Cardiac Surgery, Centre Cardiologique du Nord, 93200 Saint-Denis, France; 2 Department of Clinical and Experimental Medicine, University of Messina, 98122 Messina, Italy; 3 Department of Cardiothoracic Surgery, Weill Cornell Medicine–New York Presbyterian Medical Center, New York, NY 10065, USA; 4 Department of Cardiothoracic Surgery, Aberdeen Royal Infirmary, Aberdeen AB25 2ZN, UK

**Keywords:** SARS-CoV-2 infection, COVID-19, coronary artery thrombosis, neutrophil extracellular traps (NETs)

## Abstract

The coronavirus 2019 pandemic has affected many healthcare systems worldwide. While acute respiratory distress syndrome (ARDS) has been well-documented in COVID-19, there are several cardiovascular complications, such as myocardial infarction, ischaemic stroke, and pulmonary embolism, leading to disability and death. The link between COVID-19 and increasing thrombogenicity potentially occurs due to numerous different metabolic mechanisms, ranging from endothelial damage for direct virus infection, associated excessive formation of neutrophil extracellular traps (NETs), pathogenic activation of the renin-angiotensin-aldosterone system (RAAS), direct myocardial injury, and ischemia induced by respiratory failure, all of which have measurable biomarkers. A search was performed by interrogating three databases (MEDLINE; MEDLINE In-Process and Other Non-Indexed Citations, and EMBASE). Evidence from randomized controlled trials (RCT), prospective series, meta-analyses, and unmatched observational studies were evaluated for the processing of the algorithm and treatment of thromboembolic disease and cardiac thrombotic complications related to COVID-19 during SARS-CoV-2 infection. Studies out with the SARS-Cov-2 infection period and case reports were excluded. A total of 58 studies were included in this analysis. The role of the acute inflammatory response in the propagation of the systemic inflammatory sequelae of the disease plays a major part in determining thromboembolic disease and cardiac thrombotic complication in COVID-19. Some of the mechanisms of activation of these pathways, alongside the involved biomarkers noted in previous studies, are highlighted. Inflammatory response led to thromboembolic disease and cardiac thrombotic complications in COVID-19. NETs play a pivotal role in the pathogenesis of the inflammatory response. Despite moving into the endemic phase of the disease in most countries, thromboembolic complications in COVID-19 remain an entity that substantially impacts the health care system, with long-term effects that remain uncertain. Continuous monitoring and research are required.

## 1. Introduction

The coronavirus infection 2019 (COVID-19) occurred at the beginning of 2020, causing a devastating global pandemic and placing the health systems of high-income countries in crisis mode. We know that the morbidity and mortality associated with the development of COVID-19 are generally heralded by the onset of acute respiratory distress syndrome (ARDS) and multiorgan failure. Cardiovascular complications, such as myocardial infarction, ischaemic stroke, and pulmonary embolism (PE), may occur in patients, as well. Complications can progress to severe disabilities and even death [1,2,3,4,5,6]. Early on, during the COVID-19 pandemic, an increased frequency of arterial and venous thrombosis was recorded in many patients, which has been linked to the devastating systemic inflammation, combined with immobility and the creation of a prothrombotic context favored by the release of cytokines [7,8,9,10,11,12]. Thromboembolic disease (TED) is one of the crucial factors in causing increased cardiovascular risks in patients with COVID-19 [9,10,13,14,15,16,17,18,19,20,21].

The data revealed a high thromboembolic complication rate for patients requiring ICU admission, albeit with some variability. A total of 25% of TED cases occurred in the forms of symptomatic diseases, while 69% were diagnosed with surveillance venous ultrasound [9,14,16,17,18]. A reasonable proportion of patients developed microthrombotic processes in situ, raising a crucial key point concerning the possible genesis of endothelial damage for direct virus infection, as has been reported [22,23,24]. Some problems emerged immediately, thus complicating the clinical management of patients. In the first place, a quantification of the risk of cardiovascular complications immediately appeared very difficult because it was linked the heterogeneity of the patient population with COVID-19 to the reports of cases that included limited sample sizes, restrictions on intensive care assessments, non-homogeneous outcome definitions, and the application of different thromboprophylaxis models. Second, although recommendations for administering antithrombotic therapy to hospitalized patients with COVID-19 to prevent thromboembolic cardiovascular events were put in place early [1,7,8,9], evidence quickly emerged that a subgroup of patients experienced arterial and venous events, despite the appropriate use of thromboprophylaxis standards [1,25,26,27,28,29].

It has been suggested that patients who manifest organ dysfunction for COVID-19, due to severe SARS-CoV-2 infection disclose, an associated excessive formation of neutrophil extracellular traps (NETs) led to vascular injury. Evidence from autopsies reports highlighted the role of mechanical vessel obstruction mediated by the formation of NET aggregates as primary movers in the pathogenesis of COVID-19 [30]. Our knowledge suggests that acute cardiac injury is a common complication in patients with severe COVID-19, thus contributing to the mortality of patients with severe SARS-CoV-2 infection [3]. In these patients, the onset of ST-elevation myocardial infarction (STEMI) occurs as a severe cardiac manifestation of the disease [31]. However, the intrinsic mechanism leading to coronary thrombosis in COVID-19 has not been fully explained.

Another point of discussion is the persistence of weariness, migraine, dyspnea, heart abnormality, cognitive and attention impairments, sleep disorders, post-traumatic stress dysfunction, muscle discomfort, and concentration difficulties, which characterized the clinical symptoms in patients with long COVID-19. Although, under the molecular profile, the renin-angiotensin-aldosterone system (RAAS) is decisively involved in the pathogenesis of COVID-19 taking the stage, since the development of the acute phase of the viral infection, RAAS seems to also play a crucial role in the pathogenesis of long-term COVID, due to its action on different organs and tissues [32,33].

**Figure 1:**
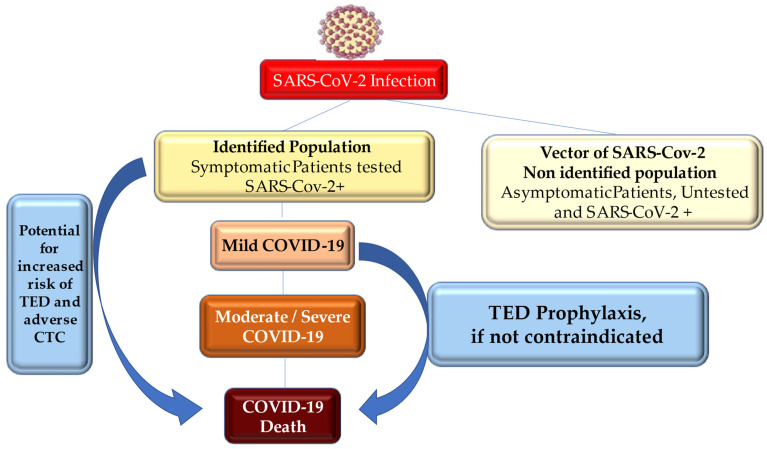
SARS-CoV-2 has spread with dissimilarity rates of the infection among population, and it has been characterized by distinct case fatality rates, across various regions and countries. Inflammatory response, increased age, and bedridden status, which are more frequently observed in severe coronavirus disease 2019 (COVID-19), may contribute to thrombosis and adverse outcomes. Abbreviations: CTC, cardiac thrombotic complication; SARS-CoV-2, severe acute respiratory syndrome-coronavirus-2; TED, thromboembolic disease.

To foster a broader understanding of cardiovascular complications that occurred in COVID-19 and provide a guide for clinicians, we discuss the current evidence base, in regard to the mechanisms that support thromboembolic disease and cardiac thrombotic complications during the spread of SARS-CoV-2 in the uninfected population and involving asymptomatic and symptomatic patients. An evidence-based algorithm for the treatment of thromboembolic disease and cardiac thrombotic complications during COVID-19 infections was processed (Figure 1).

The proposed systematic review was registered with the Open Science Framework (OSF) Registry, at the following address: https://osf.io/dm57g (accessed on 15 September 2022).

## 2. Search Method and Systematic Literature Review

**Figure 2:**
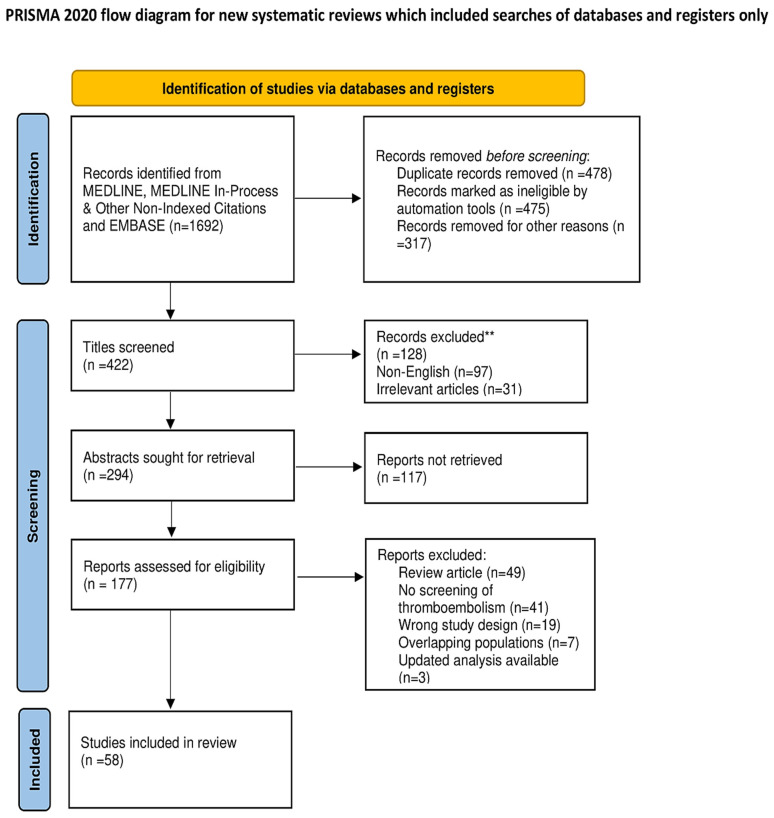
PRISMA 2020 flow diagram.

In January 2022, databases (MEDLINE; MEDLINE In-Process and Other Non-Indexed Citations, and EMBASE) were searched, using the terms “SARS-CoV-2,” “COVID-19,” “thromboembolic disease”, ” venous thromboembolic disease”, “COVID-19 venous thromboembolic”, “cardiac thrombotic complication”, “COVID-19 cardiac thrombotic complication”, “COVID-19 myocardial ischemia”, “neutrophil extracellular traps”, and “COVID-19 neutrophil extracellular traps”. For these studies, abstracts of the included manuscripts were considered and agreed upon. The present review focuses on data from randomized controlled trials (RCT), prospective series, meta-analyses, and unmatched observational studies that were considered for the processing of the algorithm for the treatment of thromboembolic disease and cardiac thrombotic complication inflammatory during SARS-CoV-2 infection. Data were extracted from the main publication, and searches were performed by two independent researchers (P.N, SSAS, using the blind method). A third independent reviewer estimated pertinence (FN). No funding was received for this study. The review was not formally registered. The protocol was not prepared. The authors have no conflicts of interest to declare. PRISMA flow diagram for systematic review and PRISMA checklist are reported in Figure 2 and Supplementary Table S1.

## 3. Results

Results focused on the pivotal mechanism of pathogenesis and transmission of SARS-CoV-2 and provided evidence insight into the hemostasis parameters and thromboembolic disease during COVID-19. Again, substantial evidence emerged regarding the role of SARS-CoV-2 in causing heart disease (Table 1).

## 4. Pathogenesis and Transmission

SARS-CoV-2 consists of a single-strand RNA coronavirus, which uses the angiotensin-converting enzyme (ACE) as the point of entry to infect human cells [66]. ACE is greatly expressed in lung alveolar cells, cardiac myocytes, the vascular endothelium, and other cells. SARS-CoV-2 expresses the glycoprotein S protein, with a peak of 180 kDa (S), which serves to identify the ACE2 receptor. Protein S is involved in cellular infection, with two crucial roles: it promotes binding to ACE2 from the amino-terminal region and promotes the fusion of viral and cell membranes across the carboxy-terminal region. Furthermore, lung cell infection is determined by the proteolytic activation of spikes that occur at a host-mediated polybasic furin cleavage site. It is important to note that the furin cleavage site is also present in the influenza A virus, but it is not expressed by SARS-CoV [67,68,69].

SARS-CoV-2 infection determines an involvement of the vascular endothelium with the associated endotheliitis, thrombosis, and marked infiltration of inflammatory cells. In patients with severe clinical evolution, a rupture of the vascular barrier and edema occur [22]. The resulting vascular angiogenesis, either intussusceptive or germinative, differentiates the pulmonary pathobiology of patients who have COVID-19 from those of severe influenza virus-related infections [24]. Immunohistochemistry revealed a high expression of ACE2 in alveolar epithelial and endothelial capillary cells in the autopsy findings of patients who died from severe forms of COVID-19. Furthermore, the presence of ACE2-positive lymphocytes, as well as the evidence of an interaction between the ACE2 receptor and immune cells, has been detected in the perivascular tissue or the alveoli of the lungs of patients infected with SARS-CoV 2 [22,23,24].

In particular, patients with COVID-19 had well-defined and characteristic pulmonary vascular alterations. Evidence from autopsy controls reported diffuse alveolar damage with the infiltration of perivascular T cells. Histopathological examinations described the severe endothelial injury associated with the presence of intracellular viruses and destroyed cell membranes. The pulmonary vessels manifest that diffused thrombosis with microangiopathy and the homogeneous accumulation of fibrin was necessary. These lesions are associated with marked interstitial edema and early interalveolar organization [22,24].

From a cytological point of view, patients who died from COVID-19 showed CD3 T cells and high levels of CD4-positive T cells in the lungs. A significant amount of CD8-positive T cells were recorded, as well as a substantial increase in neutrophils (CD15 positive) [24].

SARS-CoV-2 infection leads to an acute inflammatory stimulus, which can, among other things, destabilize the atherosclerotic plaque and induce acute myocardial infarction (AMI). A particular role during SARS-CoV-2 infection is played by the so-called “cytokine storm” [1,23]. In particular, cytokines, such as IL-1 α, IL-1β, IL-6, and TNF-α, can perturb all the protective functions of the normal endothelium and enhance the pathological processes. The pathophysiological mechanism of a cytokine storm is closely related to the self-induction of the proinflammatory cytokine IL-1. IL-1, by inducing its own gene expression, determines an amplification of its production levels, thus leading to a storm of cytokines [57,70,71]. IL-1 also induces the expression of other proinflammatory cytokines, including TNF-α and IL-1, as well as leukocyte invasion. The latter induces the production of chemotactic molecules, including chemokines, causing the penetration of inflammatory cells into the tissues [72]. Meanwhile, IL-1 stimulates the production of IL-6. IL-6 is a 27KD cytokine involved in a variety of immune and inflammatory responses; however, plasma levels of IL-6 under physiological conditions are generally very low. During acute infection, a large variety of cells, including macrophages, B, and T lymphocytes, increase the production of IL-6. Again, IL-6 offers a stimulus to the acute phase response. It induces the synthesis of fibrinogen, the precursor of clots, PAI-1 (an important inhibitor of our endogenous fibrinolytic mediators), and C-reactive protein (a biomarker of inflammation closely linked to COVID-19). During infection, the endothelium becomes activated, resulting in the loss of barrier function, expression of adhesion molecules, such as soluble ICAM-1 (intercellular adhesion molecule 1) and soluble VCAM-1 (vascular cell adhesion molecule 1), release of VWF (which allows platelet binding), the expression of TF (which activates the coagulation system) [1,23,34,35,36,37,73].

The transmission mechanism of SARS-CoV-2 is mainly mediated by the inhalation of viral particles that penetrate the respiratory tract [66]. Its contagiousness also depends on the survival of the virus for 24/72 h on the surfaces, in relation to the type of surface, which allows for the possibility of fomite transmission [69]. COVID-19 occurs with initial symptoms similar to other viral syndromes, including fever, fatigue, headache, cough, shortness of breath, diarrhea, and myalgia. Likewise, regarding the other zoonotic coronaviruses that preceded it, such as SARS and Middle East respiratory syndrome, COVID-19 also holds the peculiarity of causing severe illness, thus promoting acute respiratory disease syndrome (ARDS), systemic inflammatory response syndrome (SIRS), multiorgan implication, and shock [34]. No age group is exempt from the development of serious diseases and risk of serious complications. However, patients of older age and with comorbidities, such as cardiovascular disease, have a higher risk of developing more severe forms of the disease [35], regarding the most common laboratory changes lymphopenia and increase in lactate dehydrogenase occurring in patients with COVID-19 [73]. Again, the elevation of pro-inflammatory markers, such as C-reactive protein, D-dimer, ferritin, and interleukin-6 (IL-6), occur as prominent variations [1,15,36,37]. In particular, the levels of IL-6 can be related to the more complicated forms of the disease and presence of a procoagulant profile [1,15].

The spread of SARS-CoV-2 in the world population has taught us that there is wide international variability in the prevention measures applied, and the control strategies have been different between local authorities. A different availability in diagnostic tests and inhomogeneous access to treatment was also noted in therapeutic strategies, as well as a variability in results reported for COVID-19. Given the emergency of these concerns, we have witnessed a discrepancy between the diagnosed cases, victims, and mortality rates. Furthermore, only today are large cohorts available for extensive evaluation, so the evidence existing two years prior, including data reporting on thrombotic and cardiac complications, is based on a larger population.

## 5. Hemostasis Parameters during COVID-19

COVID-19 determines various haemostatic abnormalities; among these, the most frequent are mild thrombocytopenia [38,39,74,75] and increased levels of D-dimer [40,41,76,77]. Patients who experience this type of heamostatic abnormality develop an increased risk of being admitted to intensive care for mechanical ventilation. For these individuals, the risk of a nefarious evolution of the disease and death is substantial. Diagnostic certainty, based on other tests and related data produced, was less evident and often contradictory [39,42,77,78]. The crucial key point concerns the variability in disease severity, which is associated with the prolongation of both the prothrombin time (PT) and international normalized ratio (INR) [1,36,43]. Furthermore, an elongation of thrombin time (TT) was recorded in patients with COVID-19 [35,44,45], and they variably disclosed a trend toward noticeable changes in activated partial thromboplastin time (aPTT) [1,35,45,79]. A study published in the early stages of the pandemic spread [11] found that, in a cohort of 183 patients with COVID-19, 21 (11.5%) died. Considerable statistical evidence was disclosed between patients who died and those who survived, regarding increased levels of D-dimer and fibrin degradation produced, showing levels approximately 3.5 and 1.9 times, respectively, with a PT prolongation of 14% (*p* < 0.001). It is important to underline that, by examining data from the International Society for Thrombosis and Hemostasis (ISTH) [80] for DIC, these were in line with the mortality from DIC in deceased COVID-19 patients, which reached 71% of the total, compared to 0.6% of the survivor population. COVID-19 has taught us that these haemostatic changes can induce some forms of coagulopathy that can predispose to thrombotic events, although the complex mechanisms that favor them deserve further consideration, especially with regard to the formation of microthrombi and production of NETs (Table 2).

### Thromboembolic Disease Diagnosed in COVID-19 Patients

Platelets play a crucial role in viral infections, as has long been proven. Evidence suggesting the presence of influenza virus type A (IAV) particles was recorded in the platelets of patients with acute influenza infection. Once the IAV was embodied into the platelets, TLR7-dependent C3 was released, with the subsequent activation of neutrophils, as well as the extracellular neutrophil trap (NET) release [10,30]. Platelets interpret a substantial role in maintaining vascular integrity, but they can trigger the mechanisms that support thrombosis. More recently, the role of platelets in the immune response to viral infections has been studied, focusing on its active involvement in the host’s immune response [90,91]. The enormous amount of data available on the pathophysiological mechanisms that support SARS-CoV-2 infection has clearly disclosed that, during the viral infection, the risk of thrombosis is greater. A recent review discussed the potential role of platelets in thrombosis in COVID-19, confirming the Chinese study that found a close correlation between thrombocytopenia and risk of in-hospital mortality [46,90,91].

**Figure 3:**
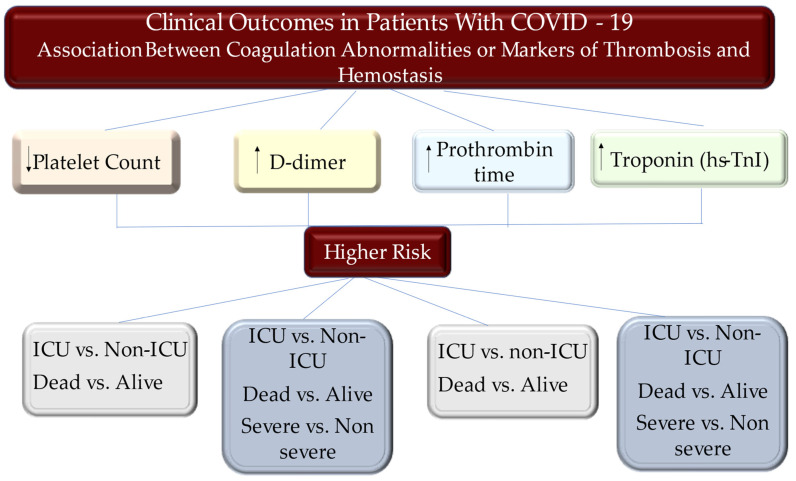
Patients who are infected with SARS-CoV-2 and develop COVID-19 experience consistent hemostatic abnormalities, of which, the most relevant comprise mild thrombocytopenia and increased D-dimer levels, which are related with a greater risk of demanding the benefit of mechanical ventilation, intensive care unit (ICU) admission, or death. Disease gravity may reveal variability of the modification of other coagulative parameters, such as a prolongation of the PT, international, and TT. The increased troponin level is associated with higher risk of STEMI and death. Abbreviations ICU, intensive care unit; PT, prothrombin time; TT, thrombin time. Ref [9,10,11,17,27,28,30,35,36,39,42,43,44,45,46,67,75,76,79,80,90,91].

Studies have reported the incidence of VTE in patients with COVID-19 [1,27,28]. A Chinese retrospective study worked in this direction by revealing a percentage, i.e., 25% (20 out of 81) of patients admitted to the ICU, in whom an accident of VTE occurred. Of note, none of the patients had been managed with the use of VTE prophylaxis drugs [37]. Klok et al., in a multicenter study that included 184 patients with severe COVID-19, revealed a percentage of 31% (95% confidence interval: 20% to 41%) of patients who developed an accident of VTE. All patients were managed with VTE prophylaxis drugs, although the authors observed underdosing in 2 of the 3 recruiting centers [9]. It cannot be ruled out that VTE may go undiagnosed and unrecognized in patients with severe COVID-19. This is an important aspect during the clinical evolution of COVID-19, as ARDS in patients with COVID-19 are potentially the source of a vicious circle involving hypoxia, pulmonary vasoconstriction, pulmonary hypertension, and right ventricular failure. The onset of pulmonary embolism is an additional clinical event that often cannot be resolved (Figure 3).

SARS-CoV-2 appears to exert haemostatic changes, both as a specific effect of the infection and consequence of the cytokine storm, which has a catalytic effect on the rate of onset of SIRS, thus confirming the observations already reported in other viral infections [46,90,91,92]. SARS-CoV-2 acts on several organs. For example, it has been shown that it can lead to severe forms of COVID-19 with cerebral infarction and bilateral limb ischemia in the setting of elevated antiphospholipid antibodies, in the context of elevated antiphospholipid antibodies. The latter plays an important role in the pathophysiology of COVID-19-associated thrombosis, as observed during the formation of microthrombi and NETs [93,94,95]. We have reported the case of a 10-day right carotid artery thrombosis, developing an intermediate grade form of COVID-19 in a 47-year-old woman with no comorbidities and negative antiphospholipid antibody assay. Regression of the thrombus was achieved after the application of the anticoagulant treatment [47]. However, further investigation deserves the study of the haemostatic changes that emerged with COVID-19, as well as a correlation both with the localization of the infection in the liver, with relative hepatic dysfunction and suspected drug-induced liver injury (DILI) [96,97]. However, Teschke et al., with Roussel Uclaf Causality Assessment Method (RUCAM)-based DILI revealed that, in COVID-19 patients with the expressed clinical characteristics, its classification as a confounding variable was well-established. So, a new correct description of the characteristics of COVID-19 is required by removing the DILI characteristics as confounding [97].

## 6. Cardiac Thrombotic Complication

Myocardial injury is a major cause of mortality from COVID. In a study performed in Wuhan, China hospitals, a high percentage of mortality (70%) was reported in patients with high cTnI levels [49]. Acute inflammation stimulus triggered by SARS-CoV-2 infection, which was embedded in atherosclerotic plaque development, with progression toward myocardial injury and acute coronary syndrome [1,2,3,4,48,49].

### 6.1. Myocardial Injury

Several studies based on COVID-19 cohort patients reported that increased troponin levels suggest poor results [49,50,98,99,100,101]. High troponin C levels are not only an expression of specific myocardial damage type 1 and 2 myocardial infarction (MI), but they are part of a broader differential diagnosis [48] that extends to non-specific myocardial damage. For example, increased troponin levels occur following renal impairment with troponin accumulation), myocarditis, and pulmonary embolism (PE) [36,100,101]. On the contrary, Lala et al. found that although myocardial damage was usual among patients who require hospitalization for a clinically critical COVID-19; however, troponin concentrations were generally recorded at low levels. Of significant impact is the increase in troponin C in COVID-19 patients with coronary artery disease (CAD). Patients with cardiovascular disease (CVD) were more likely to disclose myocardial disorder than patients without CVD. Troponin augmentation among patients hospitalized with COVID-19 was associated with a higher risk of death [48]. Concerns related to the increased level of natriuretic peptides suggest its non-specificity, while the reported pulmonary embolism events should only be included in the appropriate clinical context [102,103,104].

### 6.2. Acute Coronary Syndrome

Two independent studies have proven that myocardial injury in COVID-19 is supported by cardiac troponin levels or abnormal electrocardiography and echocardiography, whose occurrence leads to a severe form of the disease. There is also a close correlation between the more severe forms of COVID-19 with the diagnosis of higher troponin levels. [6,49]. It is important to underline that not all cases of increased levels are attributable to acute coronary syndrome (ACS), due to thrombosis. There have been several reports of patients with COVID-19 who presented with ACS caused by rupture of an atheromatous plaque (type 1 MI), thus paving the way for the role played by NETs in coronary thrombotic complications [51,52,81,105,106,107,108]. Similar cases have been reported in patients infected with the influenza virus or other viral diseases, and they have been related to a combination of several processes, such as the development of a SIRS in the presence of localized vascular or plaque inflammation [109,110,111].

Coronavirus disease 2019 (COVID-19), caused by severe acute respiratory syndrome coronavirus 2 (SARS-CoV-2), induces thrombotic disease in both the venous and arterial branches [112]. This process is orchestrated by the combination of the different players such as inflammation, platelet activation, endothelial dysfunction, and stasis. Investigators have suggested a crucial role played by extracellular neutrophil traps (NETs) in causing vascular damage and organ dysfunction in severe cases of SARS-CoV-2 infection [51,52,81,105,106,107,108,109,113]. Similarly, studies from autopsy findings have supported the role of the mechanical obstruction of vessels mediated by NET aggregates as an essential key point in the pathogenesis of COVID-19 [22,24,53,54,55,56,113,114]. Acute heart damage may occur commonly in patients with severe COVID-19, and this complication contributes to increased mortality in this cohort of individuals [113]. The event that is attributable to elevated ST myocardial infarction (STEMI) leads to a serious cardiac complication of the disease [4,31,57], and the intrinsic mechanism by which coronary thrombosis occurs in COVID-19 has been studied since the first phase of the SARS-CoV-2 pandemic [51,52].

Recently, Blasco et al. revealed evidence of NETs in coronary thrombi in patients in which COVID-19 occurred in association with STEMI. The findings explained the intrinsic mechanism that leads to coronary occlusion in patients who experienced STEMI, thus focusing on the crucial role of NETs in the pathogenesis of coronary thrombosis in the course of COVID-19 [56]. Investigators disclosed an increased level of NETS in the thrombi of all patients with COVID-19 in their thrombi. Importantly, the load of NETs was remarkably greater than in patients with STEMI and without COVID-19 infection, from the previous series reported by the same group. Evidence from immunohistochemical evaluation suggested the composition of all thrombi was constituted by a higher amount of fibrin and polymorphonuclear cells. The entirety of the lesion analyzed (thrombi, NETs, and cellular infiltrate) did not show the presence of atheromatous plaques, unlike 65% of patients in the non-infected group, who showed STEMI with evident atheromatous plaques. Furthermore, Blasco et al. found that the percentage of plaque fragments in patient historical control was close to that recorded in a previously published series of the same group 11, in which 142 patients were studied without STEMI occurring [56,57].

The work of Blasco et al. highlights some substantial key points between the presence of STEMI, NETs, and thrombus formation. We know that coagulation changes are associated with COVID-19, and these alterations suggest the presence of a hypercoagulable state that could increase the risk of thrombotic complications [93]. In patients presenting with COVID-19, the most typical modifications in blood coagulation parameters are an augmentation of D-dimer concentration, moderate reduction in platelet count, and prolongation of prothrombin time [112]. It is of note that, in the Blasco cohort, the patients with STEMI did not report any marked alteration of the coagulation parameters described above, except for one patient who presented with a high concentration of D-dimer. Likewise, this study offers a plausible explanation for the important role of neutrophils and NETs in the development of coronary thrombus in patients with COVID-19, despite the study being supported by a small sample of individuals [56].

Whether there is a cause-and-effect relationship between circulating NETs dosage and unfavorable clinical outcomes after STEMI has not yet been corroborated by solid results. Langseth et al. determined the peripherally measured NET-specific components and established a correlation with the clinical outcomes of STEMI by analyzing the serum collected, on average, 18 h after PCI. Briefly, the observational cohort study enrolled patients who received PCI (n = 956) and suffering from STEMI and were followed for a median of 4.6 years. [70] Investigators disclosed 190 events, such as heart failure hospitalization, reinfarction, unscheduled revascularization, stroke, or mortality. With the use of the serum of patients’ double-stranded DNA (dsDNA), the more specific NETs markers, i.e., myeloperoxidase-DNA and citrullinated histone, were quantified. As for the levels of NETs markers, the authors did not notably disclose a dissimilarity between the cohorts with/without a primary composite endpoint that comprised reinfarction, stroke, rehospitalization for heart failure, unscheduled revascularization >3 months after the index infarction, or all-cause death rate, to any extent, occurring first. However, dsDNA levels were higher (*p* < 0.001) in patients who died (n = 76), compared to survivors. Above-median dsDNA was associated with an increased number of deaths (54 vs. 22, *p* < 0.001), and dsDNA in the upper quartiles (Q) was associated with increased mortality. Instead, dsDNA was weakly correlated with D-dimer (rs = 0.17, *p* < 0.001), while dsDNA levels were associated with increased all-cause mortality. Again, in patients with STEMI, dsDNA was weakly correlated with hypercoagulability [112].

**Figure 4:**
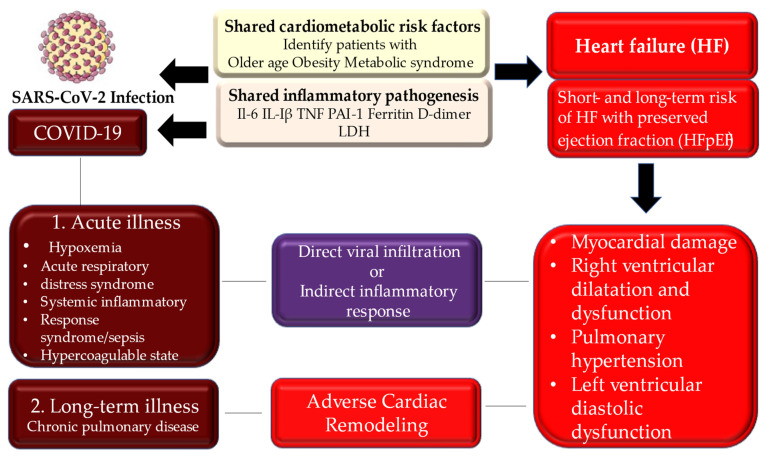
Depicts the potential intersection of acute and chronic phases of COVID-19 and risk of heart failure with preserved ejection fraction. Population at higher risk of infection and development of disease may experience an acute illness or progression toward a long-term illness. Direct viral infiltration or indirect inflammatory response, as well as adverse cardiac remodelling, can lead to chronic heart and pulmonary disease. Abbreviations: COVID-19 indicates coronavirus disease 2019; IL: interleukine; HF, heart failure; LDH, lactate dehydrogenase; PAI-1, plasminogen activator inhibitor 1; TNF, tumor necrosis factor.

Studies performed by Blasco et al. and Langseth et al. confirmed the pertinent role of NETs in the pathogenesis of SARS-CoV-2 infection. These findings sustain the concept that targeting intravascular NETs might be a relevant goal to the purpose of treatment of the patient with STEMI and represents a practicable method to prevent coronary thrombosis in patients with severe COVID-19 (Figure 4) [56,57,70].

## 7. Principles of Therapy of COVID-19 Heart Disease

### 7.1. COVID-19 and Antithrombotic Therapy in Occurring Acute Coronary Syndrome

Patients with a clinical picture consistent with ACS type 1 MI, due to plaque rupture [99], according to international guidelines (European Society of Cardiology (ESC and the American College of Cardiology (ACC)/American Heart Association (AHA)) received dual antiplatelet therapy and a therapeutic-dose anticoagulant, except for those with contraindications [115,116,117]. It is of note that the use of antithrombotic drugs for parenteral infusion does not lead to important interactions when COVID-19 experimental therapies are used [118,119,120].

Several drugs administered as antiviral agents have been used to curb COVID-19, especially to prevent the disease from evolving into more severe forms of the disease. Many of these potential drugs have not demonstrated the proposed efficacy against COVID-19. Particular attention has been paid to the clinical interactions that several of these medications have antiplatelet or anticoagulant agents. The interaction of these drugs with the coagulation system has already been studied in populations of patients who did not experience COVID-19, thus revealing that their administration can lead to both increased or reduced risks of thrombotic events or thrombocytopenia [117,118,119,120,121,122].

Among these substances, bevacizumab has been studied as a potential antiviral drug against COVID-19. Bevacizumab, while working as a monoclonal antibody with the function of interfering with vascular endothelial growth factor, however, is associated with an increased risk of adverse cardiovascular events, including myocardial infarction, cerebrovascular accidents, and VTE [119,120]. The researchers also paid attention to the drug fingolimod, which has immunomodulatory action and was tested for COVID-19; however, its primary role is to reduce reperfusion injury and improve the outcomes in patients who developed acute stroke ischemia [121]. Although hydroxychloroquine has received US Food and Drug Administration authorization for emergency use for the treatment of COVID-19, it did not have substantial proof of efficacy and safety in exercising its properties as a substance with antithrombotic action, in particular, against antiphospholipid antibodies [122].

### 7.2. Interaction between COVID-19 Investigational Therapies and Antiplatelet Medicaments

Many of the proposed therapies for the treatment of COVID-19 have revealed the existence of an interaction between these drugs and oral antiplatelet agents. Lopinavir/ritonavir was among the first drugs to be used as antiviral agents for the treatment of COVID-19. Lopinavir/ritonavir works as a protease inhibitor by interfering with the metabolism of cytochrome P450 3A4 (CYP3A4). Although clopidogrel has a specific action mediated by the active metabolite, which is mainly formed by CYP2C19. However, inhibition of CYP3A4 may also lead to a reduction in the dose efficacy of clopidogrel [118].

**Figure 5:**
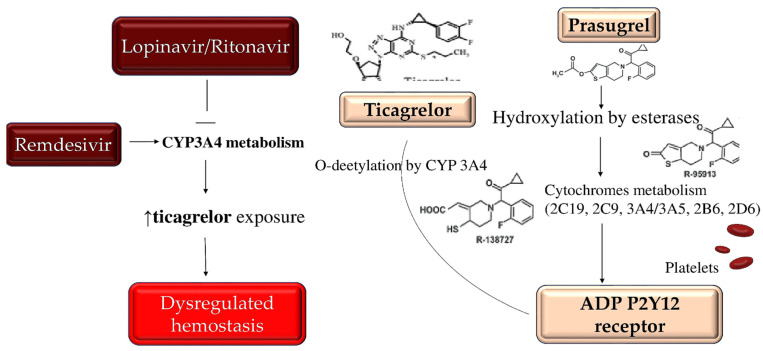
Depiction of the interaction between antiviral medicaments and antiplatelet drugs on CYP3A4 metabolism. In the marked red box, antiviral agents are represented. Lopinavir and ritonavir drive an inhibitory action on the cytochrome. This activity may increase the exposure of ticagrelor (marked yellow box), leading to a dysregulation of hemostasis (highlighted in the picture being the only depicted potential effect). Conversely, remdesivir (marked red box) is an inducer of CYP3A4 function. Differently from ticagrelor, prasugrel (marked yellow box) is metabolized by several cytochromes (2C19, 2C9, 3A4/3A5, 2B6, and 2D6), thus its effects seem to be unmodified by ritonavir or lopinavir interaction. Abbreviations: CYP3A4: cytochrome P450 3A4, ADP P2Y12 receptor: adenosine 5′diphosphate P2Y12 receptor.

Conversely, the inhibition of CYP3A4 may increase the effects of ticagrelor. Therefore, particular caution should be considered when particular antiviral drugs, such as lopinavir/ritonavir, are administered in combination with oral antiplatelet therapy. While considering the possibility of setting P2Y12-based platelet function tests to guide the use of clopidogrel or ticagrelor, however, available clinical data are scarce. The use of prasugrel, as an alternative to antiplatelet therapy, which is not prone to negative interactions of the type described, should be considered, after documenting an absence of contraindications to its use [123,124,125,126]. As for Remdesivir, which acts as a nucleotide analogue inhibitor of RNA-dependent RNA polymerase, the investigators revealed its function as an inducer of CYP3A4; however, dose adjustments of oral antiplatelet therapy are not currently recommended in conjunction with Remdesivir administration. Of substantial importance is the absence of important drug interactions between COVID-19 experimental therapies and parenteral antiplatelet agents, such as cangrelor and glycoprotein IIb/IIIa inhibitors (Figure 5) [118,123,124,125,126]. 

### 7.3. New Strategies for Patients with STEMI and Indications for the Percutaneous Coronary Intervention during COVID-19 Pandemic

The ACC and Society for Cardiovascular Angiography and Interventions delivered recommendations, regarding catheterization laboratory procedures during the SARS-CoV-2 pandemic [116]. Given the new emergency that involves the health system, due to the SARS-CoV-2 pandemic, the recommendations have been adapted by setting some criteria. Non-urgent cardiac issues were postponed, and patients were recommended to continue medical treatment to the preserve cardiovascular preparticipation evaluation (PPE). This made it possible to avoid a large waste of hospital resources, with the occupation of both hospital and intensive care beds. Furthermore, the reduced influx of patients reduced the risk of contagion by significantly minimizing exposure for both patients and healthcare professionals. In light of this, individual centers in China and elsewhere have developed adjusted ACS protocols, which call for the consideration of fibrinolytic therapy in selected patients with STEMI [1,2,3,116,117].

The first step that precedes the intervention is the achievement of an optimal diagnosis that allows distinguishing patients with non-specific myocardial damage or myocarditis from those who have symptoms attributable to true plaque rupture [127]. A useful procedure to perform a first diagnostic classification of patients, before activating the catheterization laboratory, can be the use of transthoracic echocardiography to identify wall motion abnormalities. In the presence of hospitalized patients with myocardial infarction and ST-segment elevation (STEMI), which benefit from primary percutaneous coronary intervention (PCI) because it reduces mortality and reinfarction, it is appropriate to consider the potential risk of transmission of COVID-19 from patients to healthcare professionals, or vice versa, especially in the presence of asymptomatic vectors. The changes imposed by the SARS-CoV-2 pandemic have prompted individual centers in China and many other countries to develop appropriate protocols for patients with acute coronary syndrome (ACS), which require consideration of fibrinolytic therapy in selected patients who are hospitalized with a STEMI [116,128]. Moreover, fibrinolytic therapy was the first choice in centers where timely PCI was not possible, due to conditions of severe pressure on the hospitalization system. However, for patients who reveal symptoms referable to myocarditis, in the context of COVID-19, fibrinolytic therapy should be carefully considered.

## 8. NETs and Autoantibodies May Drive COVID-19 Blood Clots: A New Investigation and Therapy Plan

Evidence emerged in severe cases of coronavirus disease 2019 (COVID-19), suggesting the occurrence of blood clots in patients who experienced pulmonary embolism with clot formation in situ, thrombosis of the deep venous veins of the lower limbs, and clot formations that led to strokes or heart attacks [30,47,81,93,94,95,106,107,108,109,113,117,129,130,131]. Zuo et al. reported that clot-promoting autoantibodies play a substantial role in causing or contributing to the development of these complications [51]. Given the previously reported evidence, the investigators announced clear parallelism between blood clotting abnormalities in patients with COVID-19 and those occurring in an autoimmune clotting disorder known as antiphospholipid syndrome (APS) [52]. The results published by Zuo et al. revealed the production of autoantibodies to phospholipids and phospholipid-binding proteins in patients who developed antiphospholipid syndrome (APS). The authors measured different types of antiphospholipid antibodies (aPL) in serum samples from 172 hospitalized patients with COVID-19. They recorded an antibody rate of 52% within the samples tested; if a stricter cutoff was used, the threshold reached 30% [51].

The significant data that distinguished this research concerns the higher levels of aPL antibodies, which were associated with the most severe forms of respiratory tract disease [30,47,51,52,93,94,95,105,129,130,131]. In addition, patients with the highest antibody titers exhibited impaired renal function and overactivity of the immune system [105]. Zuo et al. confirmed the substantial role played by extracellular neutrophil traps (NETs), which had previously been reported to be increased in COVID-19 patients [30,47,51,52,105,113]. NETs are made up by extracellular webs of chromatin, microbicidal proteins, and oxidant enzymes that are released by neutrophils to contain infections. However, when not suitably monitored, NETs are likely to spread inflammatory process and microvascular thrombosis, including in the lungs of patients with ARDS [30,47,93,94,95,113,129,130,131]. Zuo et al. revealed that increased levels of cell-free DNA, myeloperoxidase-DNA (MPO-DNA), and citrullinated histone H3 (Cit-H3) were found in the sera of COVID-19 patients; the latter two substance may be considered as NET specific markers. For the authors, this discovery paves the way for the potential clinical relevance of cell-free DNA, which is strongly correlated with the presence of specific acute phase reagents, including C-reactive protein, D-dimer, and lactate dehydrogenase, as well as the absolute neutrophil count. In particular, it was shown that MPO-DNA was associated with both cell-free DNA and absolute neutrophil counts, while Cit-H3 was related to platelet levels. A first substantial finding was conferred on both cell-free DNA and MPO-DNA, which were higher in hospitalized patients, for whom mechanical ventilation was required than in hospitalized patients who breathed room air. The second relevant evidence explaining the crucial role of NETs, associated with high levels of aPL antibodies, was related to the clot formation that occurred after injections with antibody fractions from patients with severe COVID-19. The latter led a thrombotic process more aggressively [51].

Evidence from the reports by Zuo et al. did not indicate whether these antibodies can provide a therapeutic target or be used to further define the degree of vascular damage. Furthermore, the potential degree of morbidity or mortality associated with the production of these antibodies remains to be demonstrated. Once again, the search for these antibodies can help identify patients who have COVID-19 and are most likely to benefit from aggressive anticoagulation therapy [30,47,51,52,93,94,95,105,106,107,108,113].

Working on the mechanisms that block the release of NETs, in response to these autoantibodies, could potentially be fundamental in preventing the cascade of events that produce clots in patients with COVID-19 [56]. Recently a study revealed that the antithrombotic drug dipyridamole hampers the release of NET in mice, thus supporting the drug’s potential as a treatment for APS. Another important piece of data emerging from this study supports the action of dipyridamole in reducing the release of NETs from neutrophils subject to autoantibodies in the presence of COVID-19 [132]. Dipyridamole is an inexpensive drug that exerts a safe antiplatelet action that has been shown to have immunomodulatory action associated with potential antiviral properties. Specifically, it showed a robust antiviral type I interferon immune response, which is suppressed in patients with COVID-19. Data published in a recent article have emerged that reinforce these premises [133]. The study revealed that dipyridamole suppresses SARS-CoV-2 replication, thus paving the way for a clinical trial to test its efficacy among COVID-19 hospitalized patients [134].

The evidence proven in the two studies by Zuo et al. is also of substantial relevance to other potential COVID-19 treatment strategies. Another therapy of potential interest could be plasmapheresis, which may improve outcomes among individuals with APS. It can be translated to benefit patients with COVID-19, in whom high aPL antibody titers occur [47,93,94,95]. There are two sides to the coin, so it is possible that the transfer of plasma from COVID-19 survivors and convalescent patients to seriously ill ones could also transmit the risk of impairing coagulation to them. However, plasma screening for prothrombotic autoantibodies, which was touted to potentially improve treatment, did not show the expected efficacy in clinical trials [135,136,137].

## 9. Long Term Consequences of COVID-19 Heart Disease

The affinity of SARS-CoV-2 to the host angiotensin-converting enzyme 2 receptor, as the gate of entry [58,59,60,138,139,140,141,142,143], which has been demonstrated previously for other coronaviruses, raises the probability of involving the myocardium and vascular endothelium with direct viral infection [140,141]. The cardiovascular complications of acute COVID-19 disease are well-reported; however, the post-acute cardiovascular manifestations that characterize COVID-19 that have not yet been fully elucidated deserve greater attention. In this regard, Al-Aly et al. and Xie et al. [61,144] explored the national health care database of the United States Department of Veterans Affairs, constituting a very large cohort of 153,760 individuals with COVID-19. Two groups of controls with 5,637,647 (contemporary controls) and 5,859,411 (historical controls) were studied. Interestingly, Xie et al. noted that, over the first 30 days of infection, patients with COVID-19 had an increased risk of cardiovascular disease-related events affecting several classes, which comprehended cerebrovascular disorders, arrhythmias, ischaemic and non-ischaemic heart disease, pericarditis, myocarditis, heart failure, and thromboembolic disease.

The results reported by Xie et al. [61] offered substantial clarification regarding the higher risks and charges that were shown among individuals who did not require hospitalization during the acute phase of the infection. The risk of developing a cardiovascular complication after COVID-19 gradually increased in the care setting in which patients were treated during the acute phase. In detail, non-hospitalized patients revealed a low risk, while the latter increased from hospitalized patients to those requiring the ICU. Furthermore, both the 1-year risk and burden of cardiovascular disease in acute COVID-19 survivors were considerable.

What emerges from the study by Xie et al. confirms that COVID-19 is a disease with a significant social impact. Particular attention to the care pathways of those who survive acute episodes of COVID-19 is necessary, including cardiovascular diseases [34,35,36,61,73,139].

## 10. Discussion

Several reports from series by China, Italy, and the United States reveal that most of the affected patients who develop COVID-19 experience a relatively mild clinical form; however, the clinical picture may evolve toward life-threatening disease. The evidence that emerged in the past two years supports the idea that the individuals at the highest risk of serious illness, those requiring intensive care hospitalization and at higher risk of death, are older and with underlying comorbidities, such as cardiovascular diseases [34,45,58,62,63,64,65,67,145,146]. However, even younger patients have been affected by the serious form of COVID-19, thereby requiring hospitalization. Many of these experienced a progression toward surgical intervention demand, which was complicated with nefarious evolution and death [62,63,64,65,106].

Clinical evolution of COVID-19, influenza, and other diseases supported by an acute inflammatory state may involve heart structures, and coronary artery disease may occur as a complication in this population of patients. It is of note that individuals who experience risk factors for atherosclerotic cardiovascular disease have an increased risk of acute coronary syndromes during the disease [110,111,147]. It is suggested that acute coronary events, similar to type 2 myocardial infarction, could be related to the crucial augmentation of myocardial demand that is mainly due to the infection, so that the progressive evolutive phase of this process is represented by myocardial damage or infarction. [148] However, an uncontrolled increase in the levels of circulating inflammatory cytokines that characterizes the intense systemic inflammatory activity may determine the instability or rupture of the atherosclerotic plaque. Again, in patients with the severe form of COVID-19 requiring hospitalization, heart failure complications may occur, thus suggesting that the evolution towards haemodynamic decompensation is related to the stressful condition in the presence of serious infectious diseases [62,63,64,65,106,147,148,149].

The evidence published revealed that patients with underlying cardiovascular disease were prevalently older and more prone to higher risks of adverse outcomes and death. This population of old individuals developed an aggressive form of COVID-19, sustained by a severe inflammatory state, as compared to younger patients [34,35,36,45,48,59,61,73]. It is of note that, regarding the Middle East respiratory syndrome coronavirus outbreak, several cases of acute/fulminant myocarditis with heart failure have been reported, in association with the localization of the heart infection in SARS-CoV-2 [139].

Two independent large series from Wuhan hospitals [48,59] provided circumstantial evidence, regarding the incidence and consequences of myocardial lesions in patients with SARS-CoV-2. Shi et al. explored a population of 416 hospitalized patients, with COVID-19 disclosing a rate of 19.7% of myocardial damage and increased level of troponin I (TnI) levels. In patients with myocardial damage, the hospital stay was higher, with a mortality rate of 51.2%, as compared to 4.5% in patients without myocardial damage. Furthermore, in patients who developed myocardial damage, higher levels of TnI elevation were correlated with higher mortality rates [59].

Guo et al. [48] worked on a cohort of 187 hospitalized patients with laboratory-confirmed COVID-19, reporting that 27.8% had myocardial damage with elevated troponin T (TnT) levels. The investigators focused their attention on C-reactive protein and N-terminal pro-B-type natriuretic peptide (NT-proBNP), thus providing additional novel insights. In-hospital mortality was 59.6% in patients with high TnT levels, compared to those with normal TnT levels (8.9%). Another substantial result supported those individuals with elevated TnT levels and underlying cardiovascular disease recorded the highest mortality rates of 69.4%. Mortality rates were found to be lower in patients with high TnT levels, without prior cardiovascular history. It is important to highlight that, importantly, despite an announced mortality rate of 13.3%, patients with evidence of cardiovascular disease, but without increased TnT levels, did not reveal a high mortality rate [48].

Lastly, TnT levels were notably associated with levels of C-reactive protein and N-terminal pro-B-type natriuretic peptide (NT-proBNP), thus explaining that myocardial damage was related to the severity of the inflammatory state and ventricular dysfunction. Both the TnT and NT-proBNP levels increased progressively during hospitalization in patients with evolutionary deteriorating clinical courses. Conversely, in patients in which COVID-19 occurred in a less severe form, more favorable outcomes, with lower levels of these biomarkers, were revealed [48].

Some points of convergence emerged from studies of Shi et al. and Guo et al. [48,59], who have offered substantially similar evidence in patients with COVID-19, thus disclosing elevated levels of TnI or TnT in those individuals who develop myocardial damage with adverse outcomes. The picture reveals that those patients at risk of myocardial injury present more advanced age and greater. Frequently, comorbidities resulted in increased prevalence of hypertension, coronary artery disease, heart failure, and diabetes, compared to the cohorts with normal levels of TnI or TnT. Undoubtedly a more severe systemic inflammation was found in patients with myocardial damage, including substantial increases in PMNs, higher levels of C-reactive protein, and procalcitonin. In addition, greater levels of creatine kinase, myoglobin, and NT-proBNP marked this population of patients. A finding that emerges in patients with the severe form of COVID-19, coupled with myocardial injury, is a greater acuity of the disease. Likewise, a higher incidence of acute respiratory distress syndrome and more frequent necessitation of mechanical ventilatory support, compared to those without myocardial damage, was recorded [48,59].

The studies of Shi et al. and Guo et al. were confirmed by other publications, based on cardiac autopsy and PCI performed in patients with COVID-19, reporting results that are consistent with the history of patients who experienced the severe disease. The findings here offer an explanation to the greater clinical acuity that occurred in older patients with pre-existing cardiovascular comorbidities and diabetes, who have contracted SARS-CoV-2 and are most prone to developing the severe disease. Again, this population of patients revealed an increased risk of developing myocardial damage and significantly higher short-term mortality rate [48,59].

Systemic inflammation and uncontrolled coagulopathy in COVID-19 patients play a pivotal role, and a more complete explanation has recently been offered regarding the crucial key point concerning serious SARS-CoV-2 infections, which can destabilize patients with coronary artery disease or heart failure [42,43,44,45,48,62,63,64,65,92,146]. The investigators suggested that a complementary mechanism is performed by systemic inflammatory stimuli, thus leading to greater oxygen consumption that results in demand ischemia, which evolves into myocardial damage or plaque rupture. This picture is frequent in SARS-CoV-2 infection, and it is similar to other coronaviruses, as it can elicit the intense release of multiple cytokines and chemokines [67,140]. This orchestrated process is decisive, not only in causing vascular inflammation, plaque instability, and inflammation of the myocardium, but also in triggering the release of NETs [30,47,51,52,57,105,130,131,132,133].

**Figure 6:**
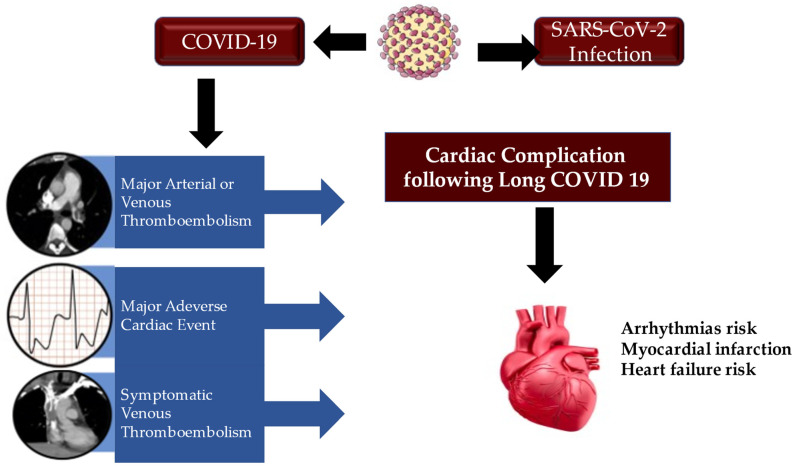
The acute clinical manifestations of COVID-19 are well-characterized by inflammatory response, endothelial dysfunction and overlapping infection that can evolve into major arterial and venous thromboembolism, major adverse cardiac event, and symptomatic venous thromboembolism. In COVID, heart condition patients may reveal a range of increased cardiovascular risks. Abbreviations: COVID-19: coronavirus disease 2019; SARS-CoV-2: severe acute respiratory syndrome-coronavirus-2.

Cases of myocarditis have been described in patients with COVID-19. Associated myocardial damage, with or without pre-existing cardiovascular comorbidities, may occur. [110] A well-documented case of acute myocarditis, following a respiratory infection, coupled with COVID-19, was described in a 53-year-old Italian woman [140]. However, several studies have been documented, focusing on potential direct viral infection of the myocardium, which is another possible modus operandi in causing myocardial damage. [60,141] Cardiac autopsies revealed that the virus was found in interstitial myocardial tissue, without the presence of replication in myocardial cells lacking unequivocal myocarditis [22,53,54,55,56] (Figure 6).

## 11. Limitations

One of the major limitations of the study involves the real-world presentation of data; hence, its inherent biases in study design. The robustness of study results were assumed during a pandemic with a higher number of observational studies included, given the acuity of disease. A large number of studies were also excluded, due to the non-English nature of these studies, which were mainly from China, the initial epicenter of the disease.

## 12. Conclusions

The deleterious effects of COVID-19 are well-documented within the literature, especially with regards to the acute phase of the illness and multiorgan involvement. However, understanding long-term COVID is a larger proposition, which may have further ramifications on healthcare resources as further evidence of chronic inflammation surfaces. Vaccinations have permitted the transition to endemicity, but COVID19’s true effects were not completely documented and understood at the time of writing.

## Figures and Tables

**Table 1:** Characteristics of the included studies. Abbreviations: DAD, diffuse alveolar damage; SOFA, Sequential Organ Failure Assessment; other abbreviations in other figures.

First Author/Year Ref	Type of Study	Number of Patients	Mean Age, years	Finding
Klok (2020) *Thromb. Res.* [9]	Retrospective Single Center (Netherlands)	184	64 (12)	Higher incidence (31%) of TED in ICU patients. VTE in 27% (95%CI 17–37%) and arterial thrombotic events in 3.7% (95%CI 0–8.2%).
Tang (2020) *J. Thromb. Haemost* [11]	Prospective Single Center Wuan (China)	183	54.1 ± 16.2	Elevated D-dimer and FDP are common in deaths with NCP fibrin degradation product (FDP) novel coronavirus pneumonia (NCP).
Cui (2020) *J. Thromb Haemost* [14]	Prospective Single Center Wuan (China)	81	59.9 (14.1)	Higher incidence of VTE (25%) in severe NCP, with poor prognosis. High-risk groups of VTE identified for increased D-dimer.
Klok (2020) *Thromb Res.* [17]	Retrospective Single Center (Netherlands)	184	64 (12)	Higher cumulative incidence of thrombotic complications in critically ill patients with NCP. Total 95% confidence interval [CI] 41–57%. Pulmonary embolism (PE) (65/75; 87%).
Lodigiani (2020) *Thromb Res.* [15]	Prospective Single Center Milan (Italy)	388	61 (55–69)	High rate of TED within 24 h of admission. High rate of positive VTE imaging suggested to improve specific thromboprophylaxis.
Middeldorp (2020) *J Thromb Haemost.* [20]	Prospective Single Center Amsterdam (Netherlands)	75	62 (10)	Higher risk of VTE in ICU patients 42% (95% CI 30–54) at 21 days.
Tang (2020) *J. Thromb Haemost* [29]	Prospective Single Center Wuan (China)	449	65.1 ± 12.0	Anticoagulant therapy, mainly using low molecular weight heparin, is associated with better prognosis. SIC criteria were relevant or D-dimer were markedly elevated.
Huang (2022) *Lancet Respir Med.* [33]	Retrospective Single Center Wuan (China)	2469	57.0 (48.0–65.0)	Within 2 years, COVID-19 survivors had longitudinal improvements in physical and mental health; however, this population had a remarkably lower health status.
Wu (2020) *JAMA* [34]	Retrospective multicenter (China)	72,314	30 to 79 years of age (87%)	Draconian measures may be considered to limit the spread of infection.
Wu (2020) *JAMA Intern. Med.* [35]	Retrospective Multicenter (China)	201	51 (43–60)	Older had greater risk of progression toward ARDS and death HR, 6.17; 95% CI, 3.26–11.67. Higher D-dimer HR, 1.02; 95% CI, 1.01–1.04.
Zhou (2020) *Lancet* [36]	Retrospective Multicenter (China)	191	56 (46–67) Non-survivor 69 (63–76) Survivor 52 (45–58)	Older age is an increased risk factors (*p* < 0.0001), as well high SOFA score and d-dimer greater than 1 μg/mL These factors can identify poor prognosis at an early stage.
Lymperaki (2022) *Medicines* [37]	Prospective Single center	199 Non COVID (60) COVID (139)	Non COVID 9–89 COVID 28–91	Biomarkers, such as vitamin B12 (*p* = 0.0029), ROS (*p* < 0.0001), and albumin (*p* = 0.046), are useful as possible prognosis tools for an early diagnosis.
Garma (2022) *Sci. Rep.* [38]	Prospective Single center	22		ACE-2 was not expressed by infected or control platelets.
Lippi (2020) *Clin. Chim. Acta.* [39]	Retrospective Multicenter (Study level meta-analysis)	1779	38–67	Low platelet count is associated with increased risk of severe disease and mortality in patients with COVID-19.
Varikasuvu (2021) *Sci. Rep.* [40]	Retrospective Multicenter (Study level meta-analysis)	Unadjusted 26,960 Adjusted 15,653	41–73	Higher D-dimer levels provide early assess COVID-19 patients at risk for disease progression and mortality outcomes.
Du (2021) *Int. J. Clin. Pract.* [41]	Retrospective Multicenter (Study level meta-analysis)	1430 Non severe COVID (1025) Severe COVID (378)	Non severe COVID 29–74 Severe COVID 41–83	Severe COVID-19 patients reveal a higher concentration of D-dimer, when compared with non-severe patients.
Han (2020) *Clin Chem Lab Med* [42]	Prospective Single Center Wuan (China)	94		Patients with SARS-CoV-2 reveal significant changes in coagulation function, as compared with healthy people. Monitoring D-dimer and FDP values may be helpful to identify severe cases.
Yang (2020) *Lancet Respir Med.* [43]	Retrospective Single Center Wuan (China)	710 52 critically	59·7 (13·3)	Older patients (>65 years) with comorbidities and ARDS are at increased risk of death.
Gao (2020) *J. Med. Virol.* [44]	Retrospective Single Center Wuan (China)	43	Severe COVID 45.20 ± 7.68 Mild COVID 42.96 ± 14.00	IL-6 and d-D tandem testing predict severity of COVID (sensitivity 93.3%, for IL-6 and 96.4%.d-D).
Wang (2020) *JAMA* [45]	Retrospective Single Center Wuan (China)	138	56 (42–68)	A total of 41% of patients with COVID-19 have presumed hospital-related transmission. A total of 26% of patients received ICU care, and mortality was 4.3%.
Yang (2020) *J. Thromb Haemost* [46]	Retrospective Single Center Wuan (China)	1476	Survivors 56 (46–65) Non survivors 67 (59–75)	Thrombocytopenia is marked in patients with COVID-19, and it is associated with increased risk of in-hospital mortality.
Nappi (2022) *J. Clin. Med.* [47]	Retrospective Multicenter (Systematic review)	38,485	(29–86)	NETs are implicated in the pathogenesis of the inflammatory response during COVID-19, and long-term effects requires ongoing monitoring and research.
Guo (2020) *JAMA cardiology* [48]	Retrospective Single Center Wuan (China)	187	58.50 (14.66)	Myocardial injury is significantly associated with fatal outcome of COVID-19 with increased cardiac dysfunction and arrhythmias.
Zhu (2021) *Immun. Inflamm. Dis.* [49]	Retrospective Multicenter (Study level meta-analysis)	15,354	40 (1–96)	Hypertension, cardiovascular disease, acute cardiac injury, and related laboratory indicators are associated with the severity of COVID-19.
Lala (2020) *JACC* [50]	Prospective single center	985	66.4 (18–100)	Myocardial injury is prevalent among patients hospitalized with COVID-19. Low levels of troponin are revealed.
Zuo (2020) *Sci. Transl. Med.* [51]	Prospective single center	172	61 ± 17 (25–95)	Patients hospitalized with COVID-19 reveal transient positivity for APL antibodies. APL autoantibodies are potentially pathogenic.
Zuo (2020) *JCI Insight* [52]	Prospective single center	80	61 ± 15 (29–91)	Sera from patients with COVID-19 disclose NET release.
Bryce et al. (2021) *Mod. Pathol* [53]	Retrospective Single center	100	68 (29 to 94)	A total of 82 cases were DAD. Hemphagocytosis, higher cytokines IL-6, IL-8, and TNFα.
Schaefer et al. (2020) *Mod. Pathol.* [54]	Retrospective Single center	7	66 (50 to 77)	A total of 5 cases diffused DAD. Two cases alveolar injuries. SARS-CoV-2 infection involving epithelial lung cell in acute phase. No endothelial cell infection.
Delorey et al. (2021) *Nature* [55]	Retrospective Single center	32	30 to 89	Higher viral RNAs in phagocytic mononuclear and endothelial lung cells. Transcriptional alterations in multiple cell types in the heart tissue.
Lindner et al. (2020) *JAMA Cardiol.* [56]	Prospective Single center	39	68 (78–89)	SARS-CoV-2 directly infects the myocardium. Absence of inflammatory cell infiltrates in patient with SARS-CoV-2 infection. Higher cytokine response.
Varga et al. (2020) *Lancet* [22]	Retrospective Single center	3	63 (58–61)	Lymphocytic endotheliitis in lung, heart, kidney, and liver. Apoptotic bodies in the heart; mononuclear cells in lung.
Ackerman et al. (2020) *N. Engl. J. Med.* [24]	Retrospective Single center	14 SARS-CoV-2 7 H1N1 7	68 ± 9.2 years (female) 80 ± 11.5 years (male)	Alveolar capillary microthrombi 9 times more in SARS-CoV-2. Higher CD3, CD4, and CD-8 positive T cells in SARS-CoV-2. Lower neutrophils (CD15).
Blasco (2020) *JAMA Cardiology* [57]	Prospective Single center	55	COVID 62 (14) Non COVID 58 (12)	In patients with COVID-19 and myocardial infarction, NETs seem to play a major role in the pathogenesis of STEMI.
Chen (2020) *Lancet* [58]	Retrospective Multicenter center Wuan (China)	99	55.5 (13.1)	The COVID-19 infection is more likely to affect older males with comorbidities, resulting in severe and even fatal acute respiratory distress syndrome.
Shi (2020) *JAMA Cardiol.* [59]	Retrospective Multicenter center Wuan (China)	416	64 (21–95)	Cardiac injury is a common evidence among hospitalized patients with COVID-19, and it is associated with higher risk of in-hospital mortality.
Szekely (2020) *Circulation* [60]	Prospective Single center	100	66.1 ± 17.2	Preservation of LV systolic function is in the majority of COVID-19 patients. Impairement of LV diastolic and RV functions. Elevated troponin and poorer clinical grade are associated with worse RV function.
Xie (2022) *Nat. Med.* [61]	Retrospective Multicenter center	153,760	61.42 (15.64)	Risk and 1-year burden of cardiovascular disease in survivors of acute COVID-19 are substantial.
Guan (2020) *NEJM* [61]	Retrospective Multicenter center Wuan (China)	1099	47 (35.0–58.0)	COVID-19 spread rapidly throughout China and caused varying degrees of illness. Many patients without fever did not have abnormal radiologic findings.
COVIDSurg Collaborative (2022) *Anaesthesia* [62]	Prospective Multicenter	128,013	55.6 (18–49)	High risk of thromboembolic complication in COVID-19 patients.
COVIDSurg Collaborative (2021) *Anaesthesia* [63]	Prospective Multicenter	96,454		Isolation before elective surgery might be associated with a small, but clinically important, increased risk of postoperative pulmonary complications.
COVIDSurg Collaborative (2021) *Br. J. Surg.* [64]	Prospective Multicenter	56,589	(18–69)	As global roll out of SARS-CoV-2 vaccination proceeds, patients needing elective surgery should be prioritized ahead of the general population.
COVIDSurg Collaborative (2021) *Anaesthesia* [65]	Prospective Multicenter	140,231	(31.4–87.4)	After a ≥7 week delay in undertaking surgery, following SARS-CoV-2 infection, patients with ongoing symptoms had a higher mortality than patients whose symptoms had resolved or who had been asymptomatic 6.0% (95% CI 3.2–8.7) vs. 2.4% (95% CI 1.4–3.4) vs. 1.3% (95% CI 0.6–2.0).

**Table 2:** The 2020 case-control retrospective studies comparing risk factors for thrombosis development in hospitalized patients with severe COVID-19 (controls) versus hospitalized patients with both severe infection and DVT or ATE (cases). VTE: venous thromboembolism, ATE: arterial thromboembolism, WBCs: white blood cells, INR: international from Nappi et al. [81], Metabolites, 25 May 2021; 11(6):341.

Authors	Total SARS-CoV-2 + Hospitalized Patients	VTE, ATE Cases	Risk Factors More Present in Cases (*p* < 0.05)	Risk Factors Similar in Cases and Controls (*p* > 0.05)	Conclusions
Stoneham et al., 2020 [82]	208	21	High WBCs, high D-dimer, high INR.	APTT ratio, fibrinogen.	Comorbidities were not associated with a higher risk of thrombosis. Monitoring of D-dimer and anti-factor Xa levels may be relevant for management.
Zuo et al., 2020 [83]	44	11	High calprotectin, markers of NETs (myeloperoxidase-DNA complexes) high D-dimer, high platelets.	Troponins, WBCs.	There was a significant difference between peak D-dimer, calprotectin and cell free DNA levels between the populations.
Zhang et al., 2020 [84]	143	66	High WBCs, older age, low oxygenation index, high rate of cardiac injury, CURB-65 score 3 to 5, Padua score ≥ 4, high D-dimer.	Platelets count.	COVID-19 is suspected to cause an additional risk factor for DVT in hospitalized patients.
Planquette et al., 2020 [85]	1042	59	High CRP, fibrinogen, d-dimer. IMV.	Comorbidities: BMI, previous VTE, ATE, cancer, hypertension, cardiovascular diseases.	No higher prevalence for VTE risk factors in cases group compared to both cases and control was found. Altered coagulation parameters were found.
Trimaille et al., 2020 [86]	289	49	High Improve score, high WBCs, d-dimer, low haemoglobin at discharge.	Padua score of 4 or more, CRP-	Lack of thromboprophylaxis is a major determinant of VTE in non-ICU COVID-19 patients. Comorbidities were not found to affect the event occurrence.
Shah et al., 2020 [87]	187	81	High troponins, ferritin, d-dimer.	Platelets count, WBCs, thromboelastography parameters.	Elevated D-dimer, ferritin, troponin and white cell count at ICU admission may reflect undiagnosed altered coagulation and be used to identify patients for CTPA.
Kolielat et al., 2020 [88]	117	18	High d-dimer, fibrinogen, ferritin.	WBCs, platelets, troponins, Il-6.	Elevated d-dimer and a less elevated fibrinogen are associated with DVT despite conventional thromboprophylactic treatment.
Kampuori et al., 2020 [89]	443	41	High d-dimer, positive Wells criteria, bilateral infiltrates on X-rays or CT scan, mechanical ventilation.	Wbcs, platelets, CRP, Padua score, Geneva score.	The combination of Wells ≥ 2 score and D−dimer ≥ 3000 ng/L is a good predictor of VTE at admission. Hospitalization in the ICU and especially mechanical ventilation were associated with VTE occurrence. The combination of Wells’ score with the D-dimer value at admission can be a useful tool to guide empiric anticoagulation therapy.

## Supplementary Materials

The following supporting information can be downloaded at: https://www.mdpi.com/article/10.3390/metabo12100889/s1, Table S1: PRISMA 2020 Checklist. Reference [150] is cited in the supplementary materials.

## Abbreviations/Acronyms

ACC	American College of Cardiology
ACE	angiotensin I-converting enzyme
ACS	acute coronary syndrome
ADP	P2Y12 receptor: adenosine 5′diphosphate P2Y12 receptor
AHA	American Heart Association
APTT	activated partial thromboplastin time
aPL	antiphospholipid
APS	antiphospholipid syndrome
ARDS	acute respiratory distress syndrome
CAD	coronary artery disease
Cit-H3	citrullinated histone H3
CTC	cardiac thrombotic complication
CVD	cardiovascular disease
COVID-19	coronavirus disease 2019
CYP3A4	cytochrome P450 3A4
CYP2C19	Cytochrome P450 2C19
CRP	C-reactive protein
DIC	disseminated intravascular coagulation
DILI	drug-induced liver injury
dsDNA	double-stranded DNA
ESC	European Society of Cardiology
ICU	intensive care unit
IL	interleukine
INR	international normalized ratio
MI	myocardial infarction
MPO-DNA	myeloperoxidase-DNA
NADPH	nicotinamide adenine dinucleotide phosphate
NETs	neutrophil extracellular traps.
PAI-1	plasminogen activator inhibitor 1
PCI	percutaneous coronary intervention
PE	pulmonary embolism
PT	prothrombin time
RAAS	renin-angiotensin-aldosterone system
RUCAM	Roussel Uclaf Causality Assessment Method
SARS-CoV-2	severe acute respiratory syndrome-coronavirus-2
SIRS	systemic inflammatory response syndrome
STEMI	ST elevation myocardial infarction
TED	thromboembolic disease
TNF	tumor necrosis factor
TT	thrombin time

## References

[1] Bikdeli B., Madhavan M.V., Jimenez D., Chuich T., Dreyfus I., Driggin E., Nigoghossian C.D., Ageno W., Madjid M., Guo Y. (2020). COVID-19 and Thrombotic or Thromboembolic Disease: Implications for Prevention, Antithrombotic Therapy, and Follow-Up: JACC State-of-the-Art Review. J. Am. Coll. Cardiol..

[2] McFadyen J.D., Stevens H., Peter K. (2020). The Emerging Threat of (Micro)Thrombosis in COVID-19 and Its Therapeutic Implications. Circ. Res..

[3] Bonow R.O., Fonarow G.C., O’Gara P.T., Yancy C.W. (2020). Association of Coronavirus Disease 2019 (COVID-19) With Myocardial Injury and Mortality. JAMA Cardiol..

[4] Jaffe A.S., Cleland J.G.F., Katus H.A. (2020). Myocardial injury in severe COVID-19 infection. Eur. Heart J..

[5] Dhakal B.P., Sweitzer N.K., Indik J.H., Acharya D., William P. (2020). SARS-CoV-2 Infection and Cardiovascular Disease: COVID-19 Heart. Heart Lung Circ..

[6] Driggin E., Madhavan M.V., Bikdeli B., Chuich T., Laracy J., Biondi-Zoccai G., Brown T.S., Der Nigoghossian C., Zidar D.A., Haythe J. (2020). Cardiovascular considerations for patients, healthcare workers, and health systems during the coronavirus disease 2019 (COVID-19) pandemic. J. Am. Coll. Cardiol..

[7] Roncon L., Zuin M., Zonzin P. (2020). Age-adjusted D-dimer cut-off levels to rule out venous thromboembolism in COVID-19 patients. Thromb. Res..

[8] Ciavarella A., Peyvandi F., Martinelli I. (2020). Where do we stand with antithrombotic prophylaxis in patients with COVID-19?. Thromb. Res..

[9] Klok F.A., Kruip M.J.H.A., Van der Meer N.J.M., Arbous M.S., Gommers D.A.M.P.J., Kant K.M., Kaptein F.H.J., van Paassen J., Stals M.A.M., Huisman M.V. (2020). Incidence of thrombotic complications in critically ill ICU patients with COVID-19. Thromb. Res..

[10] Lillicrap D. (2020). Disseminated intravascular coagulation in patients with 2019-nCoV pneumonia. J. Thromb. Haemost..

[11] Tang N., Li D., Wang X., Sun Z. (2020). Abnormal Coagulation parameters are associated with poor prognosis in patients with novel coronavirus pneumonia. J. Thromb. Haemost..

[12] Amgalan A., Othman M. (2020). Exploring possible mechanisms for COVID-19 induced thrombocytopenia: Unanswered questions. J. Thromb. Haemost..

[13] Thachil J., Tang N., Gando S., Falanga A., Levi M., Clark C., Iba T. (2020). Laboratory haemostasis monitoring in COVID-19. J. Thromb. Haemost..

[14] Cui S., Chen S., Li X., Liu S., Wang F. (2020). Prevalence of venous thromboembolism in patients with severe novel coronavirus pneumonia. J. Thromb. Haemost..

[15] Salton F., Confalonieri P., Campisciano G., Cifaldi R., Rizzardi C., Generali D., Pozzan R., Tavano S., Bozzi C., Lapadula G. (2022). Cytokine Profiles as Potential Prognostic and Therapeutic Markers in SARS-CoV-2-Induced ARDS. J. Clin. Med..

[16] Llitjos J.-F., Leclerc M., Chochois C., Monsallier J.-M., Ramakers M., Auvray M., Merouani K. (2020). High incidence of venous thromboembolic events in anticoagulated severe COVID-19 patients. J. Thromb. Haemost..

[17] Klok F.A., Kruip M.J.H.A., van der Meer N.J.M., Arbous M.S., Gommers D., Kant K.M., Kaptein F.H.J., van Paassen J., Stals M.A.M., Huisman M.V. (2020). Confirmation of the high cumulative incidence of thrombotic complications in critically ill ICU patients with COVID-19: An updated analysis. Thromb. Res..

[18] Lodigiani C., Iapichino G., Carenzo L., Cecconi M., Ferrazzi P., Sebastian T., Kucher N., Studt J.D., Sacco C., Bertuzzi A. (2020). Venous and arterial thromboembolic complications in COVID-19 patients admitted to an academic hospital in Milan, Italy. Thromb. Res..

[19] Marietta M., Ageno W., Artoni A., De Candia E., Gresele P., Marchetti M., Marcucci R., Tripodi A. (2020). COVID-19 and haemostasis: A position paper from Italian Society on Thrombosis and Haemostasis (SISET). Blood Transfus..

[20] Middeldorp S., Coppens M., van Haaps T.F., Foppen M., Vlaar A.P., Müller M.C., Bouman C.C., Beenen L.F., Kootte R.S., Heijmans J. (2020). Incidence of venous thromboembolism in hospitalized patients with COVID-19. J. Thromb. Haemost..

[21] Moll M., Zon R.L., Sylvester K.W., Chen E.C., Cheng V., Connell N.T., Fredenburgh L.E., Baron R.M., Cho M.H., Woolley A.E. (2020). VTE in ICU Patients with COVID-19. Chest.

[22] Varga Z., Flammer A.J., Steiger P., Haberecker M., Andermatt R., Zinkernagel A.S., Mehra M.R., Schuepbach R.A., Ruschitzka F., Moch H. (2020). Endothelial cell infection and endotheliitis in COVID-19. Lancet.

[23] Wichmann D., Sperhake J.P., Lütgehetmann M., Steurer S., Edler C., Heinemann A., Heinrich F., Mushumba H., Kniep I., Schröder A.S. (2020). Autopsy Findings and Venous Thromboembolism in Patients with COVID-19. Ann. Intern. Med..

[24] Ackermann M., Verleden S.E., Kuehnel M., Haverich A., Welte T., Laenger F., Vanstapel A., Werlein C., Stark H., Tzankov A. (2020). Pulmonary vascular endothelialitis, thrombosis, and angiogenesis in COVID-19. N. Engl. J. Med..

[25] Spyropoulos A.C., Levy J.H., Ageno W., Connors J.M., Hunt B.J., Iba T., Levi M., Samama C.M., Thachil J., Giannis D. (2020). Scientific and Standardization Committee communication: Clinical guidance on the diagnosis, prevention, and treatment of venous thromboembolism in hospitalized patients with COVID-19. J. Thromb. Haemost..

[26] Sofia R., Carbone M., Landoni G., Zangrillo A., Dagna L. (2022). Anticoagulation as secondary prevention of massive lung thromboses in hospitalized patients with COVID-19. Eur. J. Intern. Med..

[27] Poletto F., Spiezia L., Simion C., Campello E., Valle F.D., Tormene D., Camporese G., Simioni P. (2022). Risk Factors of Venous Thromboembolism in Noncritically Ill Patients Hospitalized for Acute COVID-19 Pneumonia Receiving Prophylactic-Dose Anticoagulation. Viruses.

[28] Moores L.K., Tritschler T., Brosnahan S., Carrier M., Collen J.F., Doerschug K., Holley A.B., Jimenez D., Le Gal G., Rali P. (2020). Prevention, diagnosis and treatment of VTE in patients with coronavirus disease 2019: CHEST guideline and expert panel report. Chest.

[29] Tang N., Bai H., Chen X., Gong J., Li D., Sun Z. (2020). Anticoagulant treatment is associated with decreased mortality in severe coronavirus disease 2019 patients with coagulopathy. J. Thromb. Haemost..

[30] Thierry A.R., Roch B. (2020). Neutrophil Extracellular Traps and By-Products Play a Key Role in COVID-19: Pathogenesis, Risk Factors, and Therapy. J. Clin. Med..

[31] Bangalore S., Sharma A., Slotwiner A., Yatskar L., Harari R., Shah B., Ibrahim H., Friedman G.H., Thompson C., Alviar C.L. (2020). ST-Segment Elevation in Patients with COVID-19—A Case Series. N. Engl. J. Med..

[32] Khazaal S., Harb J., Rima M., Annweiler C., Wu Y., Cao Z., Khattar Z.A., Legros C., Kovacic H., Fajloun Z. (2022). The Pathophysiology of Long COVID throughout the Renin-Angiotensin System. Molecules.

[33] Huang L., Li X., Gu X., Zhang H., Ren L., Guo L., Liu M., Wang Y., Cui D., Wang Y. (2022). Health outcomes in people 2 years after surviving hospitalisation with COVID-19: A longitudinal cohort study. Lancet Respir. Med..

[34] Wu Z., McGoogan J.M. (2020). Characteristics of and important lessons from the coronavirus disease 2019 (COVID-19) outbreak in China: Summary of a report of 72314 cases from the Chinese Center for Disease Control and Prevention. JAMA.

[35] Wu C., Chen X., Cai Y., Xia J., Zhou X., Xu S., Huang H., Zhang L., Zhou X., Du C. (2020). Risk Factors Associated with Acute Respiratory Distress Syndrome and Death in Patients With Coronavirus Disease 2019 Pneumonia in Wuhan, China. JAMA Intern. Med..

[36] Zhou F., Yu T., Du R., Fan G., Liu Y., Liu Z., Xiang J., Wang Y., Song B., Gu X. (2020). Clinical course and risk factors for mortality of adult in patients with COVID-19 in Wuhan, China: A retrospective cohort study. Lancet.

[37] Lymperaki E., Kazeli K., Variti G., Gerothanasi M., Gkinoudis A., Tsamesidis I., Vagdatli E. (2022). Potential Role of Certain Biomarkers Such as Vitamin B12, ROS, Albumin, as Early Predictors for Prognosis of COVID-19 Outcomes. Medicines.

[38] Garma L.D., Deng H., Goldschmidt E. (2022). Integrated analysis of transcriptomic data reveals the platelet response in COVID-19 disease. Sci. Rep..

[39] Lippi G., Plebani M., Henry B.M. (2020). Thrombocytopenia is associated with severe coronavirus disease 2019 (COVID-19) infections: A meta-analysis. Clin. Chim. Acta.

[40] Varikasuvu S.R., Varshney S., Dutt N., Munikumar M., Asfahan S., Kulkarni P.P., Gupta P. (2021). D-dimer, disease severity, and deaths (3D-study) in patients with COVID-19: A systematic review and meta-analysis of 100 studies. Sci. Rep..

[41] Du W.N., Zhang Y., Yu Y., Zhang R.M. (2021). D-dimer levels is associated with severe COVID-19 infections: A meta-analysis. Int. J. Clin. Pract..

[42] Han H., Yang L., Liu R., Liu F., Wu K.L., Li J., Liu X.H., Zhu C.L. (2020). Prominent changes in blood coagulation of patients with SARS-CoV-2 infection. Clin. Chem. Lab. Med..

[43] Yang X., Yu Y., Xu J., Shu H., Liu H., Wu Y., Zhang L., Yu Z., Fang M., Yu T. (2020). Clinical course and outcomes of critically ill patients with SARS-CoV-2 pneumonia in Wuhan, China: A single-centered, retrospective, observational study. Lancet Respir. Med..

[44] Gao Y., Li T., Han M., Li X., Wu D., Xu Y., Zhu Y., Liu Y., Wang X., Wang L. (2020). Diagnostic utility of clinical laboratory data determinations for patients with the severe COVID-19. J. Med. Virol..

[45] Wang D., Hu B., Hu C., Zhu F., Liu X., Zhang J., Wang B., Xiang H., Cheng Z., Xiong Y. (2020). Clinical Characteristics of 138 Hospitalized Patients With 2019 Novel Coronavirus—Infected Pneumonia in Wuhan, China. JAMA.

[46] Yang X., Yang Q., Wang Y., Wu Y., Xu J., Yu Y., Shang Y. (2020). Thrombocytopenia and its association with mortality in patients with COVID-19. J. Thromb. Haemost..

[47] Nappi F., Bellomo F., Avtaar Singh S.S. (2022). Insights into the Role of Neutrophils and Neutrophil Extracellular Traps in Causing Cardiovascular Complications in Patients with COVID-19: A Systematic Review. J. Clin. Med..

[48] Guo T., Fan Y., Chen M., Wu X., Zhang L., He T., Wang H., Wan J., Wang X., Lu Z. (2020). Cardiovascular Implications of Fatal Outcomes of Patients With Coronavirus Disease 2019 (COVID-19). JAMA Cardiol..

[49] Zhu Z., Wang M., Lin W., Cai Q., Zhang L., Chen D., Liu F., Xiong X., Chu J., Peng J. (2021). Cardiac biomarkers, cardiac injury, and comorbidities associated with severe illness and mortality in coronavirus disease 2019 (COVID-19): A systematic review and meta-analysis. Immun. Inflamm. Dis..

[50] Lala A., Johnson K.W., Januzzi J.L., Russak A.J., Paranjpe I., Richter F., Zhao S., Somani S., Van Vleck T., Vaid A. (2020). Prevalence and Impact of Myocardial Injury in Patients Hospitalized With COVID-19 Infection. J. Am. Coll. Cardiol..

[51] Zuo Y., Estes S.K., Ali R.A., Gandhi A.A., Yalavarthi S., Shi H., Sule G., Gockman K., Madison J.A., Zuo M. (2020). Prothrombotic autoantibodies in serum from patients hospitalized with COVID-19. Sci. Transl. Med..

[52] Zuo Y., Yalavarthi S., Shi H., Gockman K., Zuo M., Madison J.A., Blair C., Weber A., Barnes B.J., Egeblad M. (2020). Neutrophil extracellular traps in COVID-19. JCI Insight..

[53] Bryce C., Grimes Z., Pujadas E., Ahuja S., Beasley M.B., Albrecht R., Hernandez T., Stock A., Zhao Z., AlRasheed M.R. (2021). Pathophysiology of SARS-CoV-2: The Mount Sinai COVID-19 autopsy experience. Mod. Pathol..

[54] Schaefer I.M., Padera R.F., Solomon I.H., Kanjilal S., Hammer M.M., Hornick J.L., Sholl L.M. (2020). In situ detection of SARS-CoV-2 in lungs and airways of patients with COVID-19. Mod. Pathol..

[55] Delorey T.M., Ziegler C.G., Heimberg G., Normand R., Yang Y., Segerstolpe Å., Abbondanza D., Fleming S.J., Subramanian A., Montoro D.T. (2021). COVID-19 tissue atlases reveal SARS-CoV-2 pathology and cellular targets. Nature.

[56] Lindner D., Fitzek A., Bräuninger H., Aleshcheva G., Edler C., Meissner K., Scherschel K., Kirchhof P., Escher F., Schultheiss H.P. (2020). Association of Cardiac Infection With SARS-CoV-2 in Confirmed COVID-19 Autopsy Cases. JAMA Cardiol..

[57] Blasco A., Coronado M.-J., Hernández-Terciado F., Martín P., Royuela A., Ramil E., García D., Goicolea J., Del Trigo M., Ortega J. (2021). Assessment of Neutrophil Extracellular Traps in Coronary Thrombus of a Case Series of Patients With COVID-19 and Myocardial Infarction. JAMA Cardiol..

[58] Chen N., Zhou M., Dong X., Qu J., Gong F., Han Y., Qiu Y., Wang J., Liu Y., Wei Y. (2020). Epidemiological and clinical characteristics of 99 cases of 2019 novel coronavirus pneumonia in Wuhan, China: A descriptive study. Lancet.

[59] Shi S., Qin M., Shen B., Cai Y., Liu T., Yang F., Gong W., Liu X., Liang J., Zhao Q. (2020). Association of Cardiac Injury with Mortality in Hospitalized Patients With COVID-19 in Wuhan, China. JAMA Cardiol..

[60] Szekely Y., Lichter Y., Taieb P. (2020). The spectrum of cardiac manifestations in coronavirus disease 2019 (COVID-19), A systematic echocardiographic study. Circulation.

[61] Xie Y., Xu E., Bowe B., Al-Aly Z. (2022). Long-term cardiovascular outcomes of COVID-19. Nat. Med..

[62] COVIDSurg Collaborative, GlobalSurg Collaborative (2022). SARS-CoV-2 infection and venous thromboembolism after surgery: An international prospective cohort study. Anaesthesia.

[63] COVIDSurg Collaborative, GlobalSurg Collaborative (2021). Effects of pre-operative isolation on postoperative pulmonary complications after elective surgery: An international prospective cohort study. Anesthesia.

[64] COVIDSurg Collaborative, GlobalSurg Collaborative (2021). SARS-CoV-2 vaccination modelling for safe surgery to save lives: Data from an international prospective cohort study. Br. J. Surg..

[65] COVIDSurg Collaborative, GlobalSurg Collaborative (2021). Timing of surgery following SARS-CoV-2 infection: An international prospective cohort study. Anaesthesia.

[66] Walls A.C., Park Y.J., Tortorici M.A., Wall A., McGuire A.T., Veesler D. (2020). Structure, function, and antigenicity of the SARS-CoV-2 spike glycoprotein. Cell.

[67] Zhang H., Penninger J.M., Li Y., Zhong N., Slutsky A.S. (2020). Angiotensin-converting enzyme 2. (ACE2) as a SARS-CoV-2 receptor: Molecular mechanisms and potential therapeutic target. Intensive Care Med..

[68] Hoffmann M., Kleine-Weber H., Pöhlmann S. (2020). A Multibasic Cleavage Site in the Spike Protein of SARS-CoV-2 Is Essential for Infection of Human Lung Cells. Mol. Cell.

[69] Van Doremalen N., Bushmaker T., Morris D.H., Holbrook M.G., Gamble A., Williamson B.N., Tamin A., Harcourt J.L., Thornburg N.J., Gerber S.I. (2020). Aerosol and surface stability of SARS-CoV-2 as compared with SARS-CoV-1. N. Engl. J. Med..

[70] Blasco A., Bellas C., Goicolea L., Muñiz A., Abraira V., Royuela A., Mingo S., Oteo J.F., García-Touchard A., Goicolea F.J. (2017). Immunohistological Analysis of Intracoronary Thrombus Aspirate in STEMI Patients: Clinical Implications of Pathological Findings. Rev. Española De Cardiol. (Engl. Ed.).

[71] Langseth M.S., Helseth R., Ritschel V., Hansen C.H., Andersen G., Eritsland J., Halvorsen S., Fagerland M.W., Solheim S., Arnesen H. (2020). Double-Stranded DNA and NETs Components in Relation to Clinical Outcome After ST-Elevation Myocardial Infarction. Sci. Rep..

[72] Amsterdam E.A., Wenger N.K., Brindis R.G., Casey D.E., Ganiats T.G., Holmes Jr D.R., Jaffe A.S., Jneid H., Kelly R.F., Kontos M.C. (2014). 2014 AHA/ACC guideline for the management of patients with non-ST-elevation acute coronary syndromes: Executive summary: A report of the American College of Cardiology/American Heart Association Task Force on Practice Guidelines. Circulation.

[73] Zhou P., Yang X.L., Wang X.G., Hu B., Zhang L., Zhang W., Si H.R., Zhu Y., Li B., Huang C.L. (2020). A pneumonia outbreak associated with a new coronavirus of probable bat origin. Nature.

[74] Youssry I., Elaziz D.A., Ayad N., Eyada I. (2022). The Cause–Effect Dilemma of Hematologic Changes in COVID-19: One Year after the Start of the Pandemic. Hematol. Rep..

[75] Lippi G., Henry B.M., Favaloro E.J. (2021). Mean Platelet Volume Predicts Severe COVID-19 Illness. Semin. Thromb. Hemost..

[76] Lippi G., Favaloro E.J. (2020). D-dimer is associated with severity of coronavirus disease 2019 (COVID-19): A Pooled analysis. Thromb. Haemost..

[77] Thachil J., Longstaff C., Favaloro E.J., Lippi G., Urano T., Kim P.Y. (2020). the SSC Subcommittee on Fibrinolysis of the International Society on Thrombosis and Haemostasis The need for accurate D-dimer reporting in COVID-19: Communication from the ISTH SSC on fibrinolysis. J. Thromb. Haemost..

[78] Perini P., Nabulsi B., Massoni C.B., Azzarone M., Freyrie A. (2020). Acute limb ischaemia in two young, non-atherosclerotic patients with COVID-19. Lancet.

[79] Lippi G., Salvagno G.L., Ippolito L., Franchini M., Favaloro E.J. (2010). Shortened activated partial thromboplastin time: Causes and management. Blood Coagul. Fibrinolysis.

[80] Levi M.M., Toh C.H., Thachil J., Watson H.G. (2009). Guidelines for the diagnosis and management of disseminated intravascular coagulation. Br. J. Haematol..

[81] Nappi F., Iervolino A., Avtaar Singh S.S. (2021). Thromboembolic Complications of SARS-CoV-2 and Metabolic Derangements: Suggestions from Clinical Practice Evidence to Causative Agents. Metabolites.

[82] Stoneham S.M., Milne K.M., Nuttall E., Frew G.H., Sturrock B.R., Sivaloganathan H., Ladikou E.E., Drage S., Phillips B., Chevassut T.J. (2020). Thrombotic risk in COVID-19: A case series and case-control study. Clin. Med..

[83] Zuo Y., Zuo M., Yalavarthi S., Gockman K., Madison J.A., Shi H., Woodard W., Lezak S.P., Lugogo N.L., Knight J.S. (2021). Neutrophil extracellular traps and thrombosis in COVID-19. J. Thromb. Thrombolysis.

[84] Zhang L., Feng X., Zhang D., Jiang C., Mei H., Wang J., Zhang C., Li H., Xia X., Kong S. (2020). Deep Vein Thrombosis in Hospitalized Patients With COVID-19 in Wuhan, China: Prevalence, Risk Factors, and Outcome. Circulation.

[85] Planquette B., Le Berre A., Khider L., Yannoutsos A., Gendron N., de Torcy M., Mohamedi N., Jouveshomme S., Smadja D.M., Lazareth I. (2021). Prevalence and characteristics of pulmonary embolism in 1042 COVID-19 patients with respiratory symptoms: A nested case-control study. Thromb. Res..

[86] Trimaille A., Curtiaud A., Marchandot B., Matsushita K., Sato C., Leonard-Lorant I., Sattler L., Grunebaum L., Ohana M., Von Hunolstein J.J. (2020). Venous thromboembolism in non-critically ill patients with COVID-19 infection. Thromb. Res..

[87] Shah A., Donovan K., McHugh A., Pandey M., Aaron L., Bradbury C.A., Stanworth S.J., Alikhan R., Von Kier S., Maher K. (2020). Thrombotic and haemorrhagic complications in critically ill patients with COVID-19: A multicentre observational study. Crit. Care.

[88] Koleilat I., Galen B., Choinski K., Hatch A.N., Jones D.B., Billett H., Indes J., Lipsitz E. (2021). Clinical characteristics of acute lower extremity deep venous thrombosis diagnosed by duplex in patients hospitalized for coronavirus disease 2019. J. Vasc. Surg. Venous Lymphat. Disord..

[89] Kampouri E., Filippidis P., Viala B., Méan M., Pantet O., Desgranges F., Tschopp J., Regina J., Karachalias E., Bianchi C. (2020). Predicting Venous Thromboembolic Events in Patients with Coronavirus Disease 2019 Requiring Hospitalization: An Observational Retrospective Study by the COVIDIC Initiative in a Swiss University Hospital. Biomed. Res. Int..

[90] Koupenova M. (2020). Potential role of platelets in COVID-19: Implications for thrombosis. Res. Pract. Thromb. Haemost..

[91] Koupenova M., Clancy L., Corkrey H.A., Freedman J.E. (2018). Circulating Platelets as Mediators of Immunity, Inflammation, and Thrombosis. Circ. Res..

[92] Mehta P., McAuley D.F., Brown M., Sanchez E., Tattersall R.S., Manson J.J., on Behalf of theHLH Across Speciality Collaboration, UK (2020). COVID-19: Consider cytokine storm syndromes and immunosuppression. Lancet.

[93] Zhang Y., Xiao M., Zhang S., Xia P., Cao W., Jiang W., Chen H., Ding X., Zhao H., Zhang H. (2020). Coagulopathy and Antiphospholipid Antibodies in Patients with COVID-19. N. Engl. J. Med..

[94] Serrano M., Espinosa G., Serrano A., Cervera R. (2022). Antigens and Antibodies of the Antiphospholipid Syndrome as New Allies in the Pathogenesis of COVID-19 Coagulopathy. Int. J. Mol. Sci..

[95] Knight J.S., Kanthi Y. (2022). Mechanisms of immunothrombosis and vasculopathy in antiphospholipid syndrome. Semin. Immunopathol..

[96] Zhang C., Shi L., Wang F.-S. (2020). Liver injury in COVID-19: Management and challenges. Lancet Gastroenterol. Hepatol..

[97] Teschke R., Méndez-Sánchez N., Eickhoff A. (2022). Liver Injury in COVID-19 Patients with Drugs as Causatives: A Systematic Review of 996 DILI Cases Published 2020/2021 Based on RUCAM as Causality Assessment Method. Int. J. Mol. Sci..

[98] Zimmermann F.M., De Bruyne B., Pijls N.H., Desai M., Oldroyd K.G., Park S.-J., Reardon M.J., Wendler O., Woo J., Yeung A.C. (2015). Rationale and design of the Fractional Flow Reserve versus Angiography for Multivessel Evaluation (FAME) 3 Trial: A comparison of fractional flow reserve–guided percutaneous coronary intervention and coronary artery bypass graft surgery in patients with multivessel coronary artery disease. Am. Heart J..

[99] Thygesen K., Alpert J.S., Jaffe A.S., Chaitman B.R., Bax J.J., Morrow D.A., White H.D. (2018). Fourth Universal Definition of Myocardial Infarction (2018). J. Am. Coll. Cardiol..

[100] Rivara M.B., Bajwa E.K., Januzzi J.L., Gong M.N., Thompson B.T., Christiani D.C. (2012). Prognostic Significance of Elevated Cardiac Troponin-T Levels in Acute Respiratory Distress Syndrome Patients. PLoS ONE.

[101] Poe S., Vandivier-Pletsch R.H., Clay M., Wong H.R., Haynes E., Rothenberg F.G. (2015). Cardiac Troponin Measurement in the Critically Ill: Potential for Guiding Clinical Management. J. Investig. Med..

[102] Bajwa E.K., Januzzi J.L., Gong M.N., Thompson B.T., Christiani D.C. (2008). Prognostic value of plasma N-terminal probrain natriuretic peptide levels in the acute respiratory distress syndrome. Crit. Care Med..

[103] Vergaro G., Gentile F., Aimo A., Januzzi JLJr Richards A.M., Lam C.S.P., de Boer R.A., Meems L.M.G., Latini R., Staszewsky L., Anand I.S. (2022). Circulating levels and prognostic cut-offs of sST2, hs-cTnT, and NT-proBNP in women vs. men with chronic heart failure. ESC Heart Fail..

[104] Raynor A., Vallée C., Belkarfa A.-L., Lunte K., Laney M., Belhadjer Z., Vicca S., Boutten A., Bonnet D., Nivet-Antoine V. (2022). Multisystem inflammatory syndrome in children: Inputs of BNP, NT-proBNP and Galectin-3. Clin. Chim. Acta.

[105] Hampton T. (2021). Autoantibodies May Drive COVID-19 Blood Clots. JAMA.

[106] Nappi F. (2022). Incertitude Pathophysiology and Management During the First Phase of the COVID-19 Pandemic. Ann. Thorac. Surg..

[107] Nappi F., Giacinto O., Ellouze O., Nenna A., Singh S.S.A., Chello M., Bouzguenda A., Copie X. (2022). Association between COVID-19 Diagnosis and Coronary Artery Thrombosis: A Narrative Review. Biomedicines.

[108] Nappi F., Avtaar Singh S.S. (2022). Endothelial Dysfunction in SARS-CoV-2 Infection. Biomedicines.

[109] Nappi F., Iervolino A., Avtaar Singh S.S. (2022). Molecular Insights of SARS-CoV-2 Antivirals Administration: A Balance between Safety Profiles and Impact on Cardiovascular Phenotypes. Biomedicines.

[110] Madjid M., Safavi-Naeini P., Solomon S.D., Vardeny O. (2020). Potential effects of coronaviruses on the cardiovascular system: A review. JAMA Cardiol..

[111] Kwong J.C., Schwartz K.L., Campitelli M.A., Chung H., Crowcroft N.S., Karnauchow T., Katz K., Ko D.T., McGeer A.J., McNally D. (2018). Acute Myocardial Infarction after Laboratory-Confirmed Influenza Infection. N. Engl. J. Med..

[112] Corrales-Medina V., Madjid M., Musher D.M. (2010). Role of acute infection in triggering acute coronary syndromes. Lancet Infect. Dis..

[113] Levi M., Thachil J., Iba T., Levy J.H. (2020). Coagulation abnormalities and thrombosis in patients with COVID-19. Lancet Haematol..

[114] Al-Kuraishy H.M., Al-Gareeb A.I., Al-Hussaniy H.A., Al-Harcan N.A.H., Alexiou A., Batiha G.E. (2022). Neutrophil Extracellular Traps (NETs) and COVID-19: A new frontiers for therapeutic modality. Int. Immunopharmacol..

[115] Ibanez B., James S., Agewall S., Antunes M.J., Bucciarelli-Ducci C., Bueno H., Caforio A.L., Crea F., Goudevenos J.A., Halvorsen S. (2018). 2017 ESC guidelines for the management of acute myocardial infarction in patients presenting with ST segment elevation: The Task Force for the management of acute myocardial infarction in patients presenting with ST-segment elevation of the European Society of Cardiology (ESC). Eur. Heart J..

[116] Welt F.G., Shah P.B., Aronow H.D., Bortnick A.E., Henry T.D., Sherwood M.W., Young M.N., Davidson L.J., Kadavath S., Mahmud E. (2020). Catheterization laboratory considerations during the coronavirus (COVID-19) pandemic: From ACC’s Interventional Council and SCAI. J. Am. Coll Cardiol..

[117] Zeng J., Huang J., Pan L. (2020). How to balance acute myocardial infarction and COVID-19: The protocols from Sichuan Provincial People’s Hospital. Intensiv. Care Med..

[118] Cao B., Wang Y., Wen D., Liu W., Wang J., Fan G., Ruan L., Song B., Cai Y., Wei M. (2020). A Trial of Lopinavir–Ritonavir in Adults Hospitalized with Severe COVID-19. N. Engl. J. Med..

[119] Beigel J.H., Tomashek K.M., Dodd L.E., Mehta A.K., Zingman B.S., Kalil A.C., Hohmann E., Chu H.Y., Luetkemeyer A., Kline S. (2020). Remdesivir for the Treatment of COVID-19—preliminary report. N. Engl. J. Med..

[120] Totzeck M., Mincu R.I., Rassaf T. (2017). Cardiovascular Adverse Events in Patients with Cancer Treated with Bevacizumab: A Meta-Analysis of More Than 20000 Patients. J. Am. Heart Assoc..

[121] Economopoulou P., Kentepozidis N., Kotsakis A., Kapiris I. (2015). Cancer therapy and cardiovascular risk: Focus on bevacizumab. Cancer Manag. Res..

[122] Zhu Z., Fu Y., Tian D., Sun N., Han W., Chang G., Dong Y., Xu X., Liu Q., Huang D. (2015). Combination of the immune modulator fingolimod with alteplase in acute ischemic stroke: A pilot trial. Circulation.

[123] Olsen N.J., Schleich M.A., Karp D.R. (2013). Multifaceted effects of hydroxychloroquine in human disease. Semin. Arthritis Rheum..

[124] (2011). Prescribing Information. Brilinta (Ticagrelor).

[125] (2011). Product Monograph. Brilinta (Ticagrelor).

[126] Itkonen M., Tornio A., Lapatto-Reiniluoto O., Neuvonen M., Neuvonen P., Niemi M., Backman J.T. (2018). Clopidogrel Increases Dasabuvir Exposure With or Without Ritonavir, and Ritonavir Inhibits the Bioactivation of Clopidogrel. Clin. Pharmacol. Ther..

[127] Marsousi N., Daali Y., Fontana P., Reny J.-L., Ancrenaz-Sirot V., Calmy A., Rudaz S., Desmeules J.A., Samer C.F. (2018). Impact of Boosted Antiretroviral Therapy on the Pharmacokinetics and Efficacy of Clopidogrel and Prasugrel Active Metabolites. Clin. Pharmacokinet..

[128] DeFilippis E.M., Ranard L.S., Berg D.D. (2020). Cardiopulmonary Resuscitation During the COVID-19 Pandemic: A View From Trainees on the Front Line. Circulation.

[129] Valente S., Anselmi F., Cameli M. (2020). Acute coronary syndromes during COVID-19. Eur. Heart J..

[130] Fuchs T.A., Abed U., Goosmann C., Hurwitz R., Schulze I., Wahn V., Weinrauch Y., Brinkmann V., Zychlinsky A. (2007). Novel cell death program leads to neutrophil extracellular traps. J. Cell Biol..

[131] Mozzini C., Garbin U., Pasini A.M.F., Cominacini L. (2016). An exploratory look at NETosis in atherosclerosis. Intern. Emerg. Med..

[132] Almyroudis N.G., Grimm M.J., Davidson B.A., Rohm M., Urban C.F., Segal B.H. (2013). NETosis and NADPH oxidase: At the intersection of host defence, inflammation, and injury. Front. Immunol..

[133] Ali R.A., Gandhi A.A., Meng H., Yalavarthi S., Vreede A.P., Estes S.K., Palmer O.R., Bockenstedt P.L., Pinsky D.J., Greve J.M. (2019). Adenosine receptor agonism protects against NETosis and thrombosis in antiphospholipid syndrome. Nat. Commun..

[134] Liu X., Li Z., Liu S., Sun J., Chen Z., Jiang M., Zhang Q., Wei Y., Wang X., Huang Y.-Y. (2020). Potential therapeutic effects of dipyridamole in the severely ill patients with COVID-19. Acta Pharm. Sin. B.

[135] Knight J.S. Dipyridamole to Prevent Coronavirus Exacerbation of Respiratory Status (DICER) in COVID-19 (DICER). ClinicalTrials.Gov Identifier. https://clinicaltrials.gov/ct2/show/NCT04391179.

[136] Harvala H., Nguyen D., Simmonds P., Lamikanra A.A., Tsang H.P., Otter A., Maes P., Webster M., Clarkson A., Kaloyirou F. (2022). Convalescent plasma donors show enhanced cross-reactive neutralising antibody response to antigenic variants of SARS-CoV-2 following immunisation. Transfusion.

[137] Simonovich V.A., Pratx L.D.B., Scibona P., Beruto M.V., Vallone M.G., Vázquez C., Savoy N., Giunta D.H., Pérez L.G., Sánchez M.D.L. (2021). A Randomized Trial of Convalescent Plasma in COVID-19 Severe Pneumonia. N. Engl. J. Med..

[138] Lippi G., Lavie C.J., Sanchis-Gomar F. (2020). Cardiac troponin I in patients with coronavirus disease 2019 (COVID-19): Evidence from a meta-analysis. Prog. Cardiovasc. Dis..

[139] Yang C., Jin Z. (2020). An Acute Respiratory Infection Runs Into the Most Common Noncommunicable Epidemic—COVID-19 and Cardiovascular Diseases. JAMA Cardiol..

[140] Carsana L., Sonzogni A., Nasr A., Rossi R.S., Pellegrinelli A., Zerbi P., Rech R., Colombo R., Antinori S., Corbellino M. (2020). Pulmonary post-mortem findings in a large series of COVID-19 cases from Northern Italy: A two-centre descriptive study. Lancet Infect. Dis..

[141] Inciardi R.M., Lupi L., Zaccone G., Italia L., Raffo M., Tomasoni D., Cani D.S., Cerini M., Farina D., Gavazzi E. (2020). Cardiac Involvement in a Patient with Coronavirus Disease 2019 (COVID-19). JAMA Cardiol..

[142] Zou X., Chen K., Zou J., Han P., Hao J., Han Z. (2020). Single-cell RNA-seq data analysis on the receptor ACE2 expression reveals the potential risk of different human organs vulnerable to 2019-nCoV infection. Front. Med..

[143] Wan Y., Shang J., Graham R., Baric R.S., Li F. (2020). Receptor Recognition by the Novel Coronavirus from Wuhan: An Analysis Based on Decade-Long Structural Studies of SARS Coronavirus. J. Virol..

[144] Al-Aly Z., Xie Y., Bowe B. (2021). High-dimensional characterization of post-acute sequelae of COVID-19. Nature.

[145] Nappi F., Iervolino A., Singh S.A. (2021). COVID-19 Pathogenesis: From Molecular Pathway to Vaccine Administration. Biomedicines.

[146] Guan W.J., Ni Z.Y., Hu Y., Liang W.H., Qu C.Q., He J.X., Liu L., Shan H., Lei C.L., Hui D.S.C. (2020). Clinical Characteristics of coronavirus disease 2019 in China. N. Engl. J. Med..

[147] Nguyen J.L., Yang W., Ito K., Matte T.D., Shaman J., Kinney P.L. (2016). Seasonal Influenza Infections and Cardiovascular Disease Mortality. JAMA Cardiol..

[148] Smeeth L., Thomas S.L., Hall A.J., Hubbard R., Farrington P., Vallance P. (2004). Risk of Myocardial Infarction and Stroke after Acute Infection or Vaccination. N. Engl. J. Med..

[149] Oudit G.Y., Kassiri Z., Jiang C., Liu P.P., Poutanen S., Penninger J., Butany J. (2009). SARS-coronavirus modulation of myocardial ACE2 expression and inflammation in patients with SARS. Eur. J. Clin. Investig..

[150] Page M.J., McKenzie J.E., Bossuyt P.M., Boutron I., Hoffmann T.C., Mulrow C.D., Shamseer L., Tetzlaff J.M., Akl E.A., Brennan S.E. (2021). The PRISMA 2020 statement: An updated guideline for reporting systematic reviews. BMJ.

